# A Knowledge-Based Method for Association Studies on Complex Diseases

**DOI:** 10.1371/journal.pone.0044162

**Published:** 2012-09-06

**Authors:** Alireza Nazarian, Heike Sichtig, Alberto Riva

**Affiliations:** Department of Molecular Genetics and Microbiology and UF Genetics Institute, University of Florida, Gainesville, Florida, United States of America; Tor Vergata University of Rome, Italy

## Abstract

Complex disorders are a class of diseases whose phenotypic variance is caused by the interplay of multiple genetic and environmental factors. Analyzing the complexity underlying the genetic architecture of such traits may help develop more efficient diagnostic tests and therapeutic protocols. Despite the continuous advances in revealing the genetic basis of many of complex diseases using genome-wide association studies (GWAS), a major proportion of their genetic variance has remained unexplained, in part because GWAS are unable to reliably detect small individual risk contributions and to capture the underlying genetic heterogeneity. In this paper we describe a hypothesis-based method to analyze the association between multiple genetic factors and a complex phenotype. Starting from sets of markers selected based on preexisting biomedical knowledge, our method generates multi-marker models relevant to the biological process underlying a complex trait for which genotype data is available. We tested the applicability of our method using the WTCCC case-control dataset. Analyzing a number of biological pathways, the method was able to identify several immune system related multi-SNP models significantly associated with Rheumatoid Arthritis (RA) and Crohn’s disease (CD). RA-associated multi-SNP models were also replicated in an independent case-control dataset. The method we present provides a framework for capturing joint contributions of genetic factors to complex traits. In contrast to hypothesis-free approaches, its results can be given a direct biological interpretation. The replicated multi-SNP models generated by our analysis may serve as a predictor to estimate the risk of RA development in individuals of Caucasian ancestry.

## Introduction

The study of genotype-phenotype relationship in complex disorders represents a great challenge in the field of translational genetics, due to their importance from a public health perspective and to the difficulties involved in their analysis at the genetic level [1], [2]. In contrast to monogenic traits, the phenotypic variance of complex traits is caused by the interplay of multiple genetic and environmental factors [3]–[6], which limits the applicability of the traditional approaches used for mapping of Mendelian traits [1]–[3], [7], [8].

Genome-Wide Association Studies (GWAS), a powerful method for the large scale analysis of genotype-phenotype relationships [7], [9], [10], are currently the method of choice for dissecting the genetic basis of complex diseases. A large number of disorders such as cardiovascular disorders, Crohn’s disease, rheumatologic disorders, diabetes, bipolar disorder, schizophrenia and human malignancies, have thus far been studied with this approach leading to the detection of many previously undetected contributing loci (for example [1], [11]–[15]). However, despite the very large number of markers that can be genotyped using currently available array-based SNP genotyping platforms (up to one million SNP genotypes per run) [16], in most cases GWAS have so far shown limited success in explaining a considerable proportion of genetic variance of complex diseases [7], [10], [17]. This is in part due to the nature of complex diseases, and in part to limitations which are inherent to the current analytical framework employed to analyze and interpret the obtained data [18]–[20].

To start, most of the susceptibility loci so far discovered by GWAS are of small predisposing risk [4], [7] and it has been hypothesized that the genetic variance of complex diseases may be largely due to the joint contribution of multiple susceptibility loci having small individual effects [21], [22]. Detecting such infinitesimal contributions is difficult, especially when the predisposing allele is rare or the sample size is not sufficiently large [2], [3], and the very large number of markers under investigation raises the issue of multiple testing, making it even harder to reliably detect a small association signal [16]. This is also true in the case of rare variants with major phenotypic effects, as put forward by the complex disease-rare variants notion [17]. Moreover, the genetic architecture of a complex disease may include epistatic effects among interacting loci [23], [24], and effects related to gene-environment interactions [6]. Statistical methods that analyze SNPs individually are unable to address such complex effects. Although current regression-based methods have enough power to analyze some low-order interactions (e.g. - quadratic or cubic terms), provided that the non-interactive terms have significant main effects, they are unable to explore all potential combinations of markers because as the number of factors under analysis increases linearly, the number of their combinations grows exponentially, resulting in a computationally intractable situation [18], [19]. The sparsity of data and issues of overfitting and multiple testing may also impose additional hurdles in such cases [18].

A further weakness of current analytical methods is that they analyze genome-scale datasets without necessarily taking the existing biological knowledge about the trait of interest into account. It has been suggested that using prior knowledge can help reduce the dimensionality of large scale datasets, and may guide the process of extracting biologically meaningful results in a more effective manner [19], [25], [26]. Pathway-based association studies represent an example of the integration of biological knowledge with statistical data analysis, and have recently drawn attention as an alternative to hypothesis-free methods. For instance, in an attempt to extend gene set enrichment analysis used for gene expression data to GWAS, O’Dushlaine et al. [27] introduced the SNP ratio test, which calculates the ratio between the number of individually significant SNPs and the number of non-significant ones in sets of SNPs derived from every pathway of interest, and determines a pathway to be associated with the trait under investigation if its corresponding SNP ratio proves statistically significant. As another example, Peng et al. [28] and Yu et al. [29] suggested ways of computing an overall *p-*value for a gene by combining the *p-*values obtained from the association tests on all the individual SNPs belonging to that gene. Gene-level *p-*values, in turn, were employed to define a pathway-level *p-*value to investigate pathway-trait associations. However, despite the fact that the limitations of classical GWAS in the context of complex diseases are increasingly being recognized and that pathway-based association studies are receiving growing attention, there is still no optimal approach able to overcome the above described challenges and provide a comprehensive interpretation of genome-scale data.

The work presented here focuses on developing an innovative *hypothesis-based* analysis method that combines a non-deterministic computational approach to the analysis of large-scale genotype datasets with pre-existing biological and biomedical knowledge in order to increase our understanding of the genetic basis of complex disorders. In the following sections we provide a detailed description of our method, called KBAS (Knowledge-Based Association Study), and we present its application to large-scale genotyping datasets for Rheumatoid Arthritis (RA) and Crohn's disease (CD). We will use these examples to show how our method can be used to compare alternative hypotheses about the disease of interest which in turn can lead to new insights into the genetic structure of the complex disease under consideration. We also present the results of a replication study, in which we validated the results obtained on RA through a different independent dataset.

Since complex disorders are widespread and important from the public health perspective, dissecting their genetic architecture and reconstructing the integrated networks of factors underlying their pathogenesis may help develop more specific and sensitive screening and diagnostic tests and more efficient therapeutic targets [1], [19]. This in turn may lead to reducing the morbidity and mortality of these diseases [1], consequently decreasing the burden imposed on patients and society. We believe that the KBAS method will represent an advance in this direction, and will help increase our understanding of the genetic basis of complex disorders.

## Methods

### KBAS Method Overview

**Figure 1:**
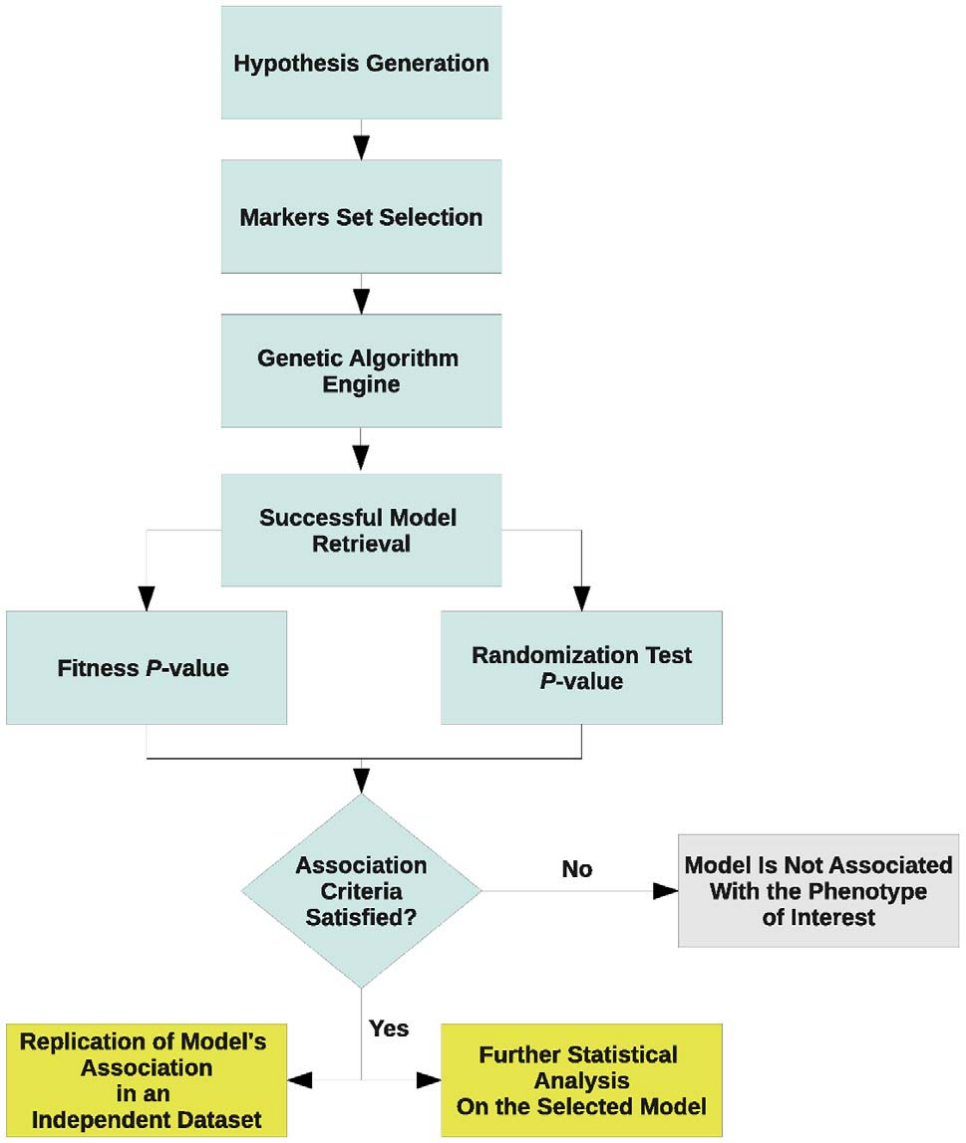
A flow chart illustrating the steps applied by the KBAS method.

We propose a *hypothesis-based* approach that performs a holistic analysis of genome-wide association data. Figure 1 illustrates the basic outline of KBAS method. Given a hypothesis formulated by the investigator, the method generates a number of *models*, consisting of sets of markers corresponding to the hypothesis under consideration. The models are then tested and refined on the basis of their ability to accurately classifying subjects as *affected* or *not affected* on the basis of their genotypes.

To start, we define an *encoding scheme* that converts the genotypes of the markers under consideration to numerical values. Each marker is then given a *weight* that quantifies its contribution to the overall genotype-phenotype association. The combination of a set of markers and their corresponding weights represents a model. The genotype values corresponding to each marker in the model, along with their respective weights, are combined according to an appropriate mathematical formula, producing a *score variable* for each subject, and the distributions of scores for the subjects in the case and control groups are then compared to evaluate the model’s classification ability. The idea at the base of our method is that, if the set of markers included in the model is relevant to the phenotype, their joint signal, obtained by combining their individual association signals into an overall variable, will be able to accurately classify cases and controls. The optimal combination of SNPs and their corresponding weights leading to a model with maximized classification ability is determined through an iterative adjustment procedure based on Genetic Algorithms (GA) which generates, tests and refines different models relevant to the hypothesis under investigation. A detailed explanation of the steps employed by KBAS method is provided in the following sections.

### Hypothesis Generation

The KBAS method we propose is aimed at verifying or disproving a hypothesis put forth by the user. In this context, the hypothesis is the statement that a particular set of genes contributes to the trait under investigation. The set of genes to be included in a hypothesis will in general be determined by the investigator on the basis of existing biological knowledge. Biochemical and regulatory pathways, gene ontology classes, gene expression databases, protein-protein interactions databases, and biomedical literature are some examples of sources of information that can be used to generate relevant hypotheses [26].

Once our hypothesis is formulated, the method tests whether a subset of genetic markers related to the specified set of genes is able to precisely separate cases from controls, when converted into a single variable through an appropriate mathematical combination of their genotype values. In the following sections we will assume that the markers under consideration are SNPs, and therefore only exhibit two alleles, but the method can be applied to any kind of polymorphic marker, given a way of encoding its genotypes into numerical values. In a typical scenario, an investigator may wish to determine which of the two different sets of genes is more likely to be involved in a disease of interest. This question can be answered by creating two competing hypotheses, each producing a set of SNPs belonging to the two gene sets, and evaluating them on the basis of their power to discriminate cases from controls.

### Genetic Algorithm

KBAS uses a Genetic Algorithm to adjust the weights of the SNPs composing a model, in order to maximize the model’s ability to accurately classify subjects into the case and control groups on the basis of the scores they receive. A Genetic Algorithm is a heuristic search algorithm able to efficiently explore very large and complex parameter spaces, in order to maximize a *fitness function* over the potential solutions of the problem under consideration [30], [31]. Genetic Algorithms belong to the class of non-deterministic computational methods, which also includes other methods such as neural networks [32], [33], genetic programming [34], and cellular automata [35]. Given the limitations of classical analysis methods, these approaches offer new strategies to address the complex issue of genotype-phenotype mapping.

A GA receives a number of randomly generated potential solutions (*organisms*) to the problem under consideration; each of which contains an encoded representation (*artificial chromosome*) of the set of parameters describing the solution. Solutions are then evaluated according to the specified fitness function, and are optimized through a simulated process of evolution using operators borrowed from evolutionary biology such as mutation, crossover and selection [30], [31]. The GA used in this work is a variation of the CHC algorithm [36], implemented by the authors. We followed the standard practice of encoding each model parameter (in this case, each SNP weight) as a binary number consisting of the appropriate number of bits. Artificial chromosomes are therefore represented by bit vectors whose length is proportional to the number of SNPs in the model. In the simplest case only one bit is used to represent each SNP’s weight: the weight therefore indicates whether the corresponding SNP is present in the model or not.

The definition of the fitness function is a critical aspect in the use of a GA. In our application the fitness of a model is a measure of the model’s ability to discriminate cases and controls, which in turn is calculated on the basis of the set of scores assigned by the corresponding model to the subjects in the case and in the control groups. The score corresponding to a particular model is defined, for each subject, as the logarithm of ratio of the conditional probabilities of the subject’s genotype under the two phenotype states of interest (e.g. diseased *vs.* healthy), according to the following formula inspired by the definition of Bayes factor [37]:
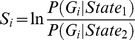
where S_i_ is the score for individual *i*, G_i_ is the genotype of individual *i* for the set of SNPs included in the model, and State_1_ and State_2_ are the two phenotype states being compared to each other, i.e. case *vs.* control.

Once the scores are computed for all subjects in the case and control groups, their distributions are compared using a two-sample t-test, and the *p-*value resulting from the comparison of the score distributions is used as the fitness measure. The assumption is that a model with high fitness generates highly different score distributions in the two groups resulting in a more extreme t-value and a smaller *p-*value compared to those generated by a model with low fitness. Therefore, models with smaller fitness *p-*values are preferred.

The GA will then rank all simulated organisms on the basis of their fitness, remove the lower half of the ranked organisms, and replace them with a new generation produced by applying the aforementioned genetic operators to the surviving organisms. Although the GA is initialized using a user-specified number of SNPs, reflecting the user’s preference for the approximate number of markers included in the model, during this step the number of non-zero weights can increase or decrease due to occurrence of crossover and mutations, causing the GA to discard some markers or to include new ones in the models. This cycle continues until a desired fitness level is attained, or the maximum number of generations is reached. Note that the desired fitness level should be adjusted according to Bonferroni’s correction [38] to account for the number of artificial chromosomes, of simulated generations, of investigated hypotheses and of pair-wise case-control comparisons performed in the analysis. At the last generation of GA, the set of weights encoded by the organism with the highest fitness is used to generate the *successful model*, that is, the one that displays the highest ability to separate cases from controls.

### Association Criteria

The successful model is then evaluated by permutation testing to validate its suggested association with the trait of interest. Holding the values of the generated score variable constant, the case-control labels of subjects in the original dataset are permuted. The model is then applied to the resulting permuted dataset and its corresponding fitness value is recorded. This process is repeated a large number of times to produce an empirical distribution of the fitness values, which can then be used to asses whether the fitness value derived from the original dataset is significant.

If the successful model shows a fitness *p-*value lower than the user-specified significance threshold and also proves significant under permutation test, it is considered to be associated with the trait under investigation and its fitness level can be used as a measure of the strength of the association. We can therefore conclude that the SNPs included in the model represent causal variants or are in linkage disequilibrium (LD) with causal variants in neighboring regions.

### Replication of the Results

Like in any association study, the significant findings should be further validated in an independent case-control dataset to prove their replicability. In the replication step, a score variable is generated and tested in the replication dataset using the same model that was generated and tested in the discovery dataset. If a model successfully discriminates cases from controls in the replication dataset and the *p-*value of its permutation test is also significant, it is considered replicated.

### Logistic Regression Analysis and Disease Risk-Score Class Diagram

To evaluate how the disease-associated successful models produced by the GA affect the risk of developing the disease under consideration, a multiple logistic regression model is fitted using the scores produced by these models as the independent variables, and the disease state (affected *vs.* healthy) as the response variable. In addition, to investigate potential interactions among the successful models under consideration, all their pairwise interaction terms are included as predictor variables as well, and a stepwise selection procedure was applied to only retain the statistically significant variables.

A simple regression model is also fitted by regressing disease state on the overall score variable computed for the entire set of SNPs present in the significant disease-associated models. To illustrate the relationship between the overall score variable and the disease risk, we then discretize the overall score variable into multiple classes and compute the posterior probability of being affected by the disease for each class of this newly generated discrete variable using Bayes’ formula [37] (see Methods S1). Logistic regression analysis is performed using SAS (v9.2).

### Method Evaluation

We used the KBAS method to test a number of hypotheses indicating that genes in a set of pathways related to the activity and regulation of the immune system play a role in the development of Rheumatoid Arthritis (RA). In addition, we tested the applicability of the KBAS method to other diseases like Crohn’s disease (CD) and type I diabetes (T1D). In particular, the results related to CD will be described in this manuscript. The tests were performed using genotype data provided by the Wellcome Trust Case-Control Consortium (WTCCC) [1]. Patients in the RA and CD groups served as cases, and healthy individuals in the 58C and NBS groups constituted the controls. Case and control groups contain ∼2000 and ∼1500 individuals respectively. Study subjects are of Caucasian ancestry, and each one was genotyped at around 500,000 SNPs using the Affymetrix platform (Affymetrix GeneChip 500 K Mapping Array). The small values of the trend test’s over-dispersion parameter (λ = 1.03 for RA and 1.11 for CD) based on principal component analysis indicates only trivial confounding effects exists related to the population stratification [1]. We converted SNP genotypes to numerical values by representing the major allele (A) at each locus as 0 and the minor allele (B) as 1. The three possible genotypes can therefore be encoded as follows: AA = 0, AB = 1, BB = 2. Alternative encoding schemes, for example to consider dominant or recessive effects, can easily be adopted.

Using two control groups provides the advantage that the successful model produced by the comparison of the case group against one of the controls (e.g. RA *vs.* NBS) can be tested in two more comparisons, one between the case group and the other control group (e.g RA *vs.* 58C), and the other one between the two control groups (58C *vs.* NBS). This increases the robustness of the inferred associations and reduces the risk of overfitting, because the successful model should be able to separate the case group from the second control group, and should not be able to separate the two control groups from each other. This in turn shows that results are reproducible and provides evidence that the GA is not simply learning to classify different groups of subjects or finding models consisting of chance aggregations of SNPs due to small differences between the groups unrelated to the disease (such as those due to geographic or ancestral factors), but produces results that are directly related to the disease of interest.

Rheumatoid arthritis is an autoimmune disorder primarily affecting joints in a monoarticular or oligoarticular pattern with subsequent progression to a polyarthritis with clinical manifestations related to inflammation of synovial membrane, and articular cartilage erosion and destruction. The etiology of the disease is unknown, but it seems both genetic susceptibility and environmental triggers contribute to the pathogenesis of the disease [39]. A number of previously reported RA-predisposing loci (for example [1], [11], [40]–[43]) implicate the role of genetic factors in the process of disease development. Crohn’s disease is also a chronic immune-mediated disorder characterized by recurrent transmural inflammation of the gastrointestinal tract [44]. It has been suggested that an inappropriate immune response to microbial flora of the intestine in genetically susceptible individuals may play an important role in the development of this disease [1], [45]. A previous meta-analysis reported over 71 CD-associated loci [15] corroborating the role of genetic factors in the disease susceptibility. The entire set of these predisposing loci accounts for about 21% of heritability of the disease [23].

Due to the suggested auto immune nature of the rheumatoid arthritis and Crohn’s disease, we focused on fourteen pathways related to different aspects of the human immune system as test-set pathways. Moreover, we selected ten other pathways involved in processes unrelated to the immune system, and therefore are likely to be irrelevant to the pathogenesis of RA and CD, to serve as negative controls in our analysis. Table 1 provides complete details on both sets of pathways. Pathway definitions were retrieved from the KEGG database (Kyoto Encyclopedia of Genes and Genomes: http://www.genome.jp/kegg/) [46].

**Table 1:** The list of pathways included in this study and their characteristics.

Pathway	KEGG ID	Number of Genes		Number of Transcripts		Number of Validated SNPs		Number of Validated SNPs in WTCCC Dataset	
**Test Pathways**									
**Antigen Processing and Presentation**	map04612	69		111		7,950		148	
**B-cell Receptor Signaling**	map04662	75		141		30,791		1,054	
**Chemokine Signaling**	map04062	190		314		71,371		2,576	
**Complement and Coagulation Cascades**	map04610	69		137		15,415		673	
**Cytokine-Cytokine Receptor Interaction**	map04060	265		453		47,551		1,811	
**Fc Epsilon RI Signaling**	map04664	79		131		34,966		1,247	
**Fc Gamma R-mediated Phagocytosis**	map04666	95		182		48,187		1,842	
**Immune Network for IgA Production**	map04672	46		61		7,538		132	
**Leukocyte Trans-endothelial Migration**	map04670	116		215		53,296		1,930	
**Natural Killer Cell Mediated Cytotoxicity**	map04650	132		236		37,095		1,262	
**Phagosome**	map04145	151		261		32,421		1,029	
**Regulation of Autophagy**	map04140	34		45		5,342		160	
**T-cell Receptor Signaling**	map04660	108		205		38,035		1,334	
**Toll-like Receptor Signaling**	map04620	102		172		19,755		585	
**Control Pathways**									
**Cardiac Muscle Contraction**	map04260		73		145		33,043		1,430
**Gap Junction**	map04540		90		148		57,854		2,257
**Glycolysis/Gluconeogenesis**	map00010		64		116		10,570		392
**Insulin Signaling**	map04910		137		245		44,633		1,289
**Nucleotide Excision Repair**	map03420		44		63		10,096		319
**Oxidative Phosphorylation**	map00190		116		175		15,100		478
**Purine Metabolism**	map00230		161		296		80,663		2,845
**Pyrimidine Metabolism**	map00240		99		160		28,338		724
**Renin Angiotensin System**	map04614		17		29		3,720		125
**Spliceosome**	map03040		126		180		14,922		474

Test-set contains immune system related pathways selected based on the preexisting knowledge about the pathogenesis of diseases under investigation and control-set contains pathways which are not likely to be relevant to the pathogenesis of the diseases of interest based on the preexisting knowledge.

For each pathway, we selected all validated SNPs found within the transcripts encoded by the genes belonging to it, extending up to 3,000 bp upstream and downstream. We then filtered this set of SNPs to retain only those that were genotyped in the WTCCC study. To further reduce the number of selected SNPs, the investigator has the option to choose a subset of “representative” SNPs from each transcript (e.g. only one SNP), on the basis of a prioritizing rule that preferentially selects non-synonymous coding SNPs, followed by promoter SNPs, exon-intron junction SNPs, synonymous coding SNPs, other exonic SNPs, and intronic SNPs. Table S1 summarizes the functional roles of the genotyped and validated SNPs related to the pathways under investigation. All procedures related to SNP set creation, manipulation and filtering were performed using Genephony, a user-friendly web-based browser for large-scale genomic datasets manipulation and for knowledge-based discovery tasks [47].

Finally, to test if the association detected for RA-associated pathways can be generalized to other cohorts, we tested the significance of the corresponding successful models in an independent case-control dataset of white Americans (NARAC) [41]. This dataset contains genotype data for 908 patients suffering from RA as the case group (NARAC-A) and for 1,260 healthy individuals as controls (NARAC-C). All subjects are of Caucasian ancestry and were genotyped at 545,080 SNPs using the Illumina Infinium HumanHap550 array. The set of SNPs genotyped in this dataset is not the same as that of the WTCCC study, due to the different genotyping platforms used. Therefore, we relied on linkage disequilibrium between markers in close proximity to replace SNPs in the successful models that were not genotyped in NARAC with the closest SNPs for which NARAC data is available.

### The KBAS Software

The KBAS method described here was implemented in a freely available software package, that can be downloaded at http://genome.ufl.edu/rivalab/KBAS/as a 64bit GNU/Linux command-line executable. The program allows users to specify the input genotype datasets (case and control groups), and the GA parameters such as number of organisms, number of generations, weights encoding scheme, etc. It also provides users with an interface to the Genephony system [47] to automatically generate sets of SNPs related to the hypotheses under study on the basis of biological knowledge. The output of the program is a file containing the names of the markers in the successful model with their respective weights and functional roles. The program can also perform randomization testing on the successful model and write the resulting *p-*value on the output file. All results described in the remainder of this paper were generated using the KBAS software.

## Results

### Analysis of the Rheumatoid Arthritis (RA) Dataset

For each of the twenty four initial sets of SNPs derived from the pathways listed in Table 1, KBAS initialized a GA using a population of 200 candidate models (represented by artificial chromosomes, using a single bit for each SNP weight). Each artificial chromosome was initialized to contain non-zero weights for a random subset of approximately 10 SNPs. The population was then evolved over 500 simulated generations, and the artificial chromosome providing the best fitness value in the final generation was used to generate the successful model.

Tables 2 and 3 summarize the *p-*values associated with the fitness of each successful model on the basis of the pairwise comparisons between the case group and the two control groups (i.e. RA *vs.* 58C and RA *vs.* NBS). Note that the significance level was adjusted to 6.944×10^−9^ according to Bonferroni’s correction, given the number of artificial chromosomes (n = 200), simulated generations (n = 500), investigated pathways (n = 24) and pair-wise population comparisons (n = 3). To validate the significance of theses fitness values, we assessed the goodness-of-fit of each model using permutation testing performed over 100,000 rounds. Given the total number of pair-wise comparisons (n = 48), we used a corrected significance threshold of 0.00104 according to Bonferroni’s correction. A randomization test *p-*value between 0.05 and 0.00104 was considered borderline. A model was considered significant if in both RA *vs.* 58C and RA *vs.* NBS comparisons had significant *p-*values, and was considered non-significant if resulted in non-significant *p-*values in at least one of the two comparisons. In all other situations, it was considered borderline. The *p-*values obtained from permutation tests are also shown in Tables 2 and 3 next to the fitness *p-*values.

**Table 2:** The *p-*values associated with the pairwise comparisons of the RA group and the two control groups using the successful models derived from immune system related pathways.

Pathway	58C *vs.* NBS	RA *vs.* 58C		RA *vs.* NBS	
	Fitness	Fitness	Randomization-test	Fitness	Randomization-test
**Antigen Processing and Presentation**	0.00589	**1.03**×**10^−9^**	**<10^−5^**	**3.96**×**10^−16^**	**<10^−5^**
**B-cell Receptor Signaling**	0.04550	**4.44**×**10^−14^**	**<10^−5^**	**5.79**×**10^−13^**	**10^−5^**
**Chemokine Signaling**	>0.05	**9.76**×**10^−11^**	**0.00073**	**1.23**×**10^−9^**	**0.00336**
**Complement and Coagulation Cascades**	>0.05	**1.19**×**10^−23^**	**<10^−5^**	**2.31**×**10^−20^**	**<10^−5^**
**Cytokine-Cytokine Receptor Interaction**	0.04981	**1.09**×**10^−20^**	**<10^−5^**	**1.64**×**10^−22^**	**<10^−5^**
**Fc Epsilon RI Signaling**	0.03246	6.97×10**^−^** ^5^	>0.05	2.47×10**^−^** ^5^	>0.05
**Fc Gamma R-mediated Phagocytosis**	0.04356	1.91×10**^−^** ^6^	>0.05	1.43×10**^−^** ^7^	0.01143
**Immune Network for IgA Production**	0.00408	**6.65**×**10^−11^**	**<10^−5^**	**4.31**×**10^−18^**	**<10^−5^**
**Leukocyte Trans-endothelial Migration**	>0.05	**1.11**×**10^−17^**	**<10^−5^**	**2.71**×**10^−18^**	**<10^−5^**
**Natural Killer Cell Mediated Cytotoxicity**	0.00437	**2.55**×**10^−9^**	**0.00263**	**7.97**×**10^−10^**	**0.00085**
**Phagosome**	>0.05	**2.48**×**10^−16^**	**<10^−5^**	**4.34**×**10^−17^**	**<10^−5^**
**Regulation of Autophagy**	>0.05	0.00941	>0.05	0.01714	>0.05
**T-cell Receptor Signaling**	0.04116	**5.83**×**10^−8^**	**0.00111**	**8.03**×**10^−8^**	**0.00161**
**Toll-like Receptor Signaling**	>0.05	**6.82**×**10^−14^**	**<10^−5^**	**2.68**×**10^−12^**	**<10^−5^**

The fitness *p-*values measure the fitness of each successful model retrieved by Genetic Algorithm engine. They are calculated by comparing original case and control datasets using corresponding successful models. Randomization-test *p-*values measure the significance of fitness *p-*values of their corresponding successful model by comparing permuted case and control datasets. According to Bonferroni’s correction, a fitness *p-*value <6.944×10**^−^** ^9^ and a randomization test *p-*value <0.00104 were considered significant. The *p-*values of the models showing strong or moderate association with rheumatoid arthritis are in bold.

**Table 3:** The *p-*values associated with the pairwise comparisons of the RA group and the two control groups using successful models derived from negative control pathways.

Pathway	58C *vs.* NBS	RA *vs.* 58C		RA *vs.* NBS	
	Fitness	Fitness	Randomization-test	Fitness	Randomization-test
**Cardiac Muscle Contraction**	>0.05	0.01392	>0.05	0.01158	>0.05
**Gap Junction**	>0.05	0.00051	>0.05	0.00202	>0.05
**Glycolysis/Gluconeogenesis**	>0.05	0.0007	>0.05	0.00288	>0.05
**Insulin Signaling**	>0.05	4.30×10**^−^** ^5^	>0.05	0.000533	>0.05
**Nucleotide Excision Repair**	>0.05	0.00144	>0.05	0.00084	>0.05
**Oxidative Phosphorylation**	0.02171	9.12×10**^−^** ^8^	0.04476	1.26×10**^−^** ^6^	>0.05
**Purine Metabolism**	>0.05	>0.05	>0.05	>0.05	>0.05
**Pyrimidine Metabolism**	>0.05	0.00782	>0.05	0.03497	>0.05
**Renin Angiotensin System**	>0.05	0.00273	>0.05	0.02638	>0.05
**Spliceosome**	0.02692	4.47×10**^−^** ^6^	>0.05	0.00024	>0.05

### Test-set Pathways

For each of the tested pathways, with the exception of the *Fc epsilon RI signaling, Fc gamma R-mediated phagocytosis, regulation of autophagy and T-cell receptor signaling* pathways, KBAS was able to identify a model that classifies cases and controls with a highly significant *p-*value, ranging from 2.55×10**^−^** ^9^ to 1.19×10**^−^** ^23^ for the RA *vs.* 58C comparisons and from 1.23×10**^−^** ^9^ to 1.64×10**^−^** ^22^ for the RA *vs.* NBS comparisons. By contrast, the successful models were unable to separate the NBS and 58C groups, and the *p-*values of the comparisons were always non-significant (see Table 2). The fact that successful models derived from these ten pathways were able to separate case-control groups with fitnesses more extreme than the pre-determined significance threshold comparing RA *vs.* NBS and RA *vs.* 58C, but were not able to discriminate the two control groups from each other, indicates that they represent sets of SNPs which could potentially be associated with rheumatoid arthritis.

In permutation tests performed by comparing RA *vs.* 58C and RA *vs.* NBS, the fitness *p-*values related to all these ten models except for those related to the *chemokine signaling* and the *natural killer cell mediated cytotoxicity* pathways, were validated with *p-*values less than 10**^−^** ^5^, suggesting their remarkable performance in distinguishing the RA cohort from unaffected individuals. The eight pathways that give rise to significant *p-*values in permutation test are therefore considered to be strongly associated with rheumatoid arthritis.

Despite the high fitness values of the models obtained from the c*hemokine signaling* and n*atural killer cell mediated cytotoxicity* pathways, permutation testing suggested only moderate association of these models with rheumatoid arthritis. While permutation testing of the model derived from the first pathway resulted in a significant *p-*value for RA *vs.* 58C comparison (*p*<7.3×10**^−^** ^4^) and a borderline *p-*value for RA *vs.* NBS comparison (*p*<3.36×10**^−^** ^3^), the *p-*values resulting from permutation testing of the model from the second pathway were borderline for RA *vs.* 58C comparison (*p*<2.63×10**^−^** ^3^) and significant for RA *vs.* NBS comparison (*p*<08.5×10**^−^** ^4^) respectively.

The successful model from the *T-cell receptor signaling* pathway yielded statistically borderline fitness values when comparing RA against 58C and NBS (*p<*5.83×10**^−^** ^8^ and *p*<8.03×10**^−^** ^8^ respectively). Once again the comparison between the two control groups had a non-significant *p-*value. In permutation testing, the original case-control *p-*values appeared to be of borderline statistical significance for both the RA *vs.* 58C comparison (*p*<1.11×10**^−^** ^3^) and the RA *vs.* NBS comparison (*p*<1.61×10**^−^** ^3^). We suggest the model derived from this pathway may also be in moderate association with RA.

Finally, the successful models derived from the *Fc epsilon RI signaling, Fc gamma R-mediated phagocytosis* and *regulation of autophagy* pathways are not considered to be in association with RA because they did not produce significant fitness *p-*values and the *p-*values resulting from the corresponding permutation tests were non-significant as well.

### Negative Control Pathways

On all pathways in the negative control set, the method failed to produce a model capable of classifying cases and controls with a statistically significant fitness *p-*value. In addition, the results of all permutation tests were non-significant (*p-*values >0.00104) (see Table 3). In three cases namely *insulin signaling, oxidative phosphorylation* and *spliceosome* pathways the fitness *p-*values related to the selected successful models were closer to the significance threshold than those of other control pathways. However, the fact that neither of their corresponding permutation tests was statistically significant confirms the poor goodness-of-fit of these models in discriminating cases from controls. These results are consistent with our prior expectation that these negative control pathways may not be relevant to the pathogenesis of rheumatoid arthritis, and do not therefore lead to a significant separation of the case and control groups.

Overall, these results indicate that the final models generated by KBAS are not just simple classifiers capable of learning the differences between two arbitrary groups of individuals. Instead, their classification power is a function of the biological relevance of criterion used to select the initial sets of SNPs: SNP sets derived form pathways that are more biologically relevant to the trait of interest will lead to more accurate and powerful models. This feature is specially important when one aims to test and rank alternative hypotheses about the trait under consideration.

### RA *vs.* CTR

We further evaluated the reproducibility of the observed associations by comparing the RA group versus the pooled population of the two control groups, here called CTR, using the successful models retrieved by GA. All the eleven models showing strong or moderate association with RA in the previous step along with model related to the *Fc gamma R-mediated phagocytosis* pathway were statistically associated to rheumatoid arthritis (see Table 4) in this analysis. Complete details regarding the RA *vs.* CTR analysis are provided in Results S1.

**Table 4:** The *p-*values associated with the comparison of the RA and CTR groups and NARAC-A and NARAC-C using the successful models derived from pathways under consideration.

Pathway	RA *vs.* CTR		NARAC-A *vs.* NARAC-C	
	Fitness	Randomization-test	Fitness	Randomization-test
**Test Pathways**				
**Antigen Processing and Presentation**	**8.17**×**10^−17^**	**<10^−5^**	**1.47**×**10 ^−17^**	**<10^−5^**
**B-cell Receptor Signaling**	**8.31**×**10^−18^**	**<10^−5^**	4.54×10**^−^** ^5^	>0.05
**Chemokine Signaling**	**1.44**×**10^−13^**	**<10^−5^**	**6.47**×**10^−8^**	**0.03463**
**Complement and Coagulation Cascades**	**3.11**×**10^−28^**	**<10^−5^**	**7.17**×**10^−25^**	**<10^−5^**
**Cytokine-Cytokine Receptor Interaction**	**5.51**×**10^−30^**	**<10^−5^**	6.34×10**^−^** ^5^	>0.05
**Fc Epsilon RI Signaling**	2.73×10**^−^** ^6^	>0.05	7.52×10**^−^** ^7^	>0.05
**Fc Gamma R-mediated Phagocytosis**	**9.09**×**10^−10^**	**3**×**10^−4^**	**5.65**×**10^−17^**	**<10^−5^**
**Intestinal Immune Network for IgA Production**	**5.99**×**10^−19^**	**<10^−5^**	**1.41**×**10^−17^**	**<10^−5^**
**Leukocyte Trans-endothelial Migration**	**4.61**×**10^−25^**	**<10^−5^**	**4.44**×**10^−9^**	**0.00310**
**Natural Killer Cell Mediated Cytotoxicity**	**7.37**×**10^−13^**	**<10^−5^**	**5.15**×**10^−9^**	**0.00793**
**Phagosome**	**2.34**×**10^−23^**	**<10^−5^**	0.00532	>0.05
**Regulation of Autophagy**	0.00172	>0.05	9.99×10**^−^** ^5^	>0.05
**T-cell Receptor Signaling**	**2.90**×**10^−10^**	**2** ×**10^−5^**	**5.85**×**10^−15^**	**<10^−5^**
**Toll-like Receptor Signaling**	**2.96**×**10^−18^**	**<10^−5^**	1.96×10**^−^** ^4^	>0.05
**Control Pathways**				
**Cardiac Muscle Contraction**	0.00405	>0.05	4.44×10**^−^** ^5^	>0.05
**Gap Junction**	7.72×10**^−^** ^5^	0.04995	0.01804	>0.05
**Glycolysis/Gluconeogenesis**	0.00025	>0.05	3.51×10**^−^** ^5^	>0.05
**Insulin Signaling**	2.37×10**^−^** ^6^	0.00860	0.00186	>0.05
**Nucleotide Excision Repair**	7.01×10**^−^** ^5^	>0.05	0.01216	>0.05
**Oxidative Phosphorylation**	1.72×10**^−^** ^9^	0.00460	2.77×10**^−^** ^6^	>0.05
**Purine Metabolism**	0.04224	>0.05	>0.05	>0.05
**Pyrimidine Metabolism**	0.00371	>0.05	0.00034	>0.05
**Renin Angiotensin System**	0.00216	>0.05	0.00248	>0.05
**Spliceosome**	1.09×10**^−^** ^6^	0.02772	5.78×10**^−^** ^6^	>0.05

According to Bonferroni’s correction, a fitness *p-*value <6.944×10**^−^** ^9^ and a randomization test *p-*value <0.00208 were considered significant. The *p-*values of the models showing significant association with rheumatoid arthritis comparing RA *vs.* CTR are in bold. Of these 12 pathways, five were replicated in NARAC dataset at the significance level of 0.00208 and three were replicated at the significance level of 0.05. The *p*-values of these replicated models are also in bold.

Table S2 shows the list of selected SNPs in the successful models derived from pathways displaying strong or moderate association with RA, the genes and chromosomes they belong to, their positions on their respective chromosome, and their functional roles. The number of SNPs in the successful models ranged from three to eight, which in all cases is less than 1% of the total number of SNPs in the corresponding initial SNP-sets (see Table 1). This indicates that KBAS was able to efficiently cope with the complexity of a very large multi-dimensional search space.

Out of 72 total SNPs in these 12 models, two of them lie within the MHC region on 6p21 (chr 6: 29,910,021–33,498,585) which is a known hot spot for several autoimmune disorders. *rs9272346* is a promoter SNP related to the HLA-DQA1 gene which was previously shown to be associated with juvenile chronic arthritis [48]. On the other hand *rs2072633,* located in an intron of the CFB gene, is neither associated with RA based on previous findings nor in LD with other RA-associated SNPs according to the HapMap LD data (International HapMap Project: http://hapmap.ncbi.nlm.nih.gov).

None of the other SNPs present in the successful models or the genes to which these SNPs belong is among RA-associated loci in previously published genome-wide association studies. There is also no evidence of linkage-disequilibrium between theses SNPs and other RA-associated SNPs according to the HapMap LD data. This indicates that the results generated by KBAS are not simply a rediscovery of strongly associated individual SNPs. Instead, our method is able to elucidate the joint contribution of previously unknown sets of SNPs to the genetic architecture of rheumatoid arthritis.

As seen in Table S2, the final models for seven of the tested pathways contained pairs of SNPs on the same chromosome. SNPs in these pairs are not in linkage disequilibrium with each other according to HapMap LD data. This shows that there is no redundancy in the selected SNPs, and therefore all the SNPs identified by the method were retained in their respective successful models.

### Multiple Logistic Regression Analysis

To evaluate the impact of the twelve successful models previously concluded to be in association with RA on the risk of being affected by the disease, and to investigate potential interactions among them, a multiple logistic regression model was fitted by regressing the disease state on the score variables produced by comparing RA *vs.* CTR using each of these twelve models, along with their pairwise interaction terms (66 terms).

The multivariate model obtained from the stepwise selection procedure contained the score variables related to all twelve pathways except for those related to the *Antigen processing and presentation* and *Phagosome* pathways. Moreover, the interaction terms of the interaction between the *cytokine-cytokine receptor interaction* and l*eukocyte trans-endothelial migration* pathways and between the *cytokine-cytokine receptor interaction* and *T-cell receptor signaling* pathways were also kept in the fitted regression model.

The fitted regression model had overall model *p-*values smaller than 10**^−^** ^4^, and the covariates of the included terms were statistically significant with *p-*values smaller than 0.0028. All covariates were positively correlated with the disease susceptibility with odds ratios between 1.652 and 2.859, except for the two aforementioned interaction terms which were negatively correlated with odds ratios of 0.266 and 0.182 respectively. The c-statistics for the model was 0.687 and the *p-*value of Hosmer*-*Lemeshow test for the fitted model was non-significant (*p*>0.05), indicating model’s goodness-of-fit. Table 5 summarizes these results.

**Table 5:** Multivariate regression of disease-state on the score variables derived from the successful models showing strong or moderate association with rheumatoid arthritis (comparing RA *vs.* CTR).

Test of Overall Model								
Test			Chi-square	df	*P-*value			
**Likelihood Ratio Test**			529.5161	12	<0.0001			
**Score Test**			497.9300	12	<0.0001			
**Wald Test**			451.4576	12	<0.0001			
**Test of Parameters**								
**Parameter**	**Parameter Estimate**	**Standard Error**	**Wald’s Chi-square**	**df**	***P-*** **value**	**Odds Ratio Estimates**		
						**Point Estimate**	**95% Confidence Interval**	
**Intercept**	-0.3790	0.0312	147.7942	1	<0.0001	-	-	-
**Pathway 1**	0.5020	0.1304	14.8224	1	0.0001	1.652	1.279	2.133
**Pathway 2**	0.6809	0.1516	20.1816	1	<0.0001	1.976	1.468	2.659
**Pathway 3**	0.8802	0.0990	79.0971	1	<0.0001	2.411	1.986	2.928
**Pathway 4**	0.7601	0.0984	59.6341	1	<0.0001	2.139	1.763	2.594
**Pathway 5**	1.0191	0.1973	26.6887	1	<0.0001	2.771	1.882	4.078
**Pathway 6**	0.7184	0.1224	34.4668	1	<0.0001	2.051	1.614	2.607
**Pathway 7**	0.6218	0.1108	31.4872	1	<0.0001	1.862	1.499	2.314
**Pathway8**	1.0506	0.1575	44.5086	1	<0.0001	2.859	2.100	3.893
**Pathway 9**	0.5614	0.1840	9.3069	1	0.0023	1.753	1.222	2.514
**Pathway 10**	0.5519	0.1274	18.7555	1	<0.0001	1.737	1.353	2.229
Pathway 4 * Pathway 7	-1.3250	0.3206	17.0822	1	<0.0001	0.266	0.142	0.498
Pathway 4 * Pathway 9	-1.7018	0.5694	8.9334	1	0.0028	0.182	0.060	0.557
**Goodness-of-fit Test**								
**Test**			**Chi-square**	**df**	***P-*** **value**			
**Hosmer - Lemeshow Test**			6.9178	8	0.5455			

Pathway 1: B-cell Receptor Signaling Pathway.

Pathway 2: Chemokine Signaling Pathway.

Pathway 3: Complement and Coagulation Cascades Pathway.

Pathway 4: Cytokine -Cytokine Receptor Interaction Pathway.

Pathway 5: Fc Gamma R-mediated Phagocytosis Pathway.

Pathway 6: Intestinal Immune Network for IgA Production Pathway.

Pathway 7: Leukocyte Trans-endothelial Migration Pathway.

Pathway 8: Natural Killer Cell Mediated Cytotoxicity Pathway.

Pathway 9: T-cell Receptor Signaling Pathway.

Pathway 10: Toll-like Receptor Signaling Pathway.

### Replication of the Results

To test if the detected significant association between the twelve immune system related pathways and RA can be generalized to other cohorts, the scores related to each of theses successful models were calculated in the NARAC dataset, and their distributions were compared between case (NARAC-A) and control (NARAC-C) groups. After performing 100,000 rounds of permutation testing over each of the models, the significance of the primary fitness *p-*value was determined and used as the replication criteria. The significance thresholds for interpreting the results were the same as used for the RA *vs.* CTR analysis.

As seen in Table 4, five out of the 12 immune-system related pathways showing moderate or strong association with RA in WTCCC dataset were replicated with fitness *p-*values ranging from 5.85×10**^−^** ^15^ to 7.17×10**^−^** ^25^ and permutation test *p-*values less than 10**^−^** ^5^. In addition, three models derived from *chemokine signaling, leukocyte trans-endothelial migration* and *natural killer cell mediated cytotoxicity* pathways resulted in permutation test *p-*values of 0.03463, 0.0031 and 0.00793 respectively, and therefore can be considered replicated at the significance level of 0.05. None of the models derived from the remaining 16 pathways gave rise to a significant *p-*value in the permutation tests. Due to the different genotyping platforms, a number of SNPs present in the successful models were replaced by new ones to conduct the replication study, as explained in Method Evaluation section. Table S3 summarizes the list of the original and substituted SNPs present in the eight replicated models. The substituted SNPs are in the range of 39 bps to 23,342 bps away from the original ones.

### Simple Logistic Regression Analysis and Disease Risk-Score Class Diagram

The disease state was also regressed on the overall score variable computed based on the all 44 SNPs present in the replicated models, to evaluate the applicability of this single score variable in predicting the disease risk. Further details are provided in Results S1 and Tables S4 and S5.

**Figure 2:**
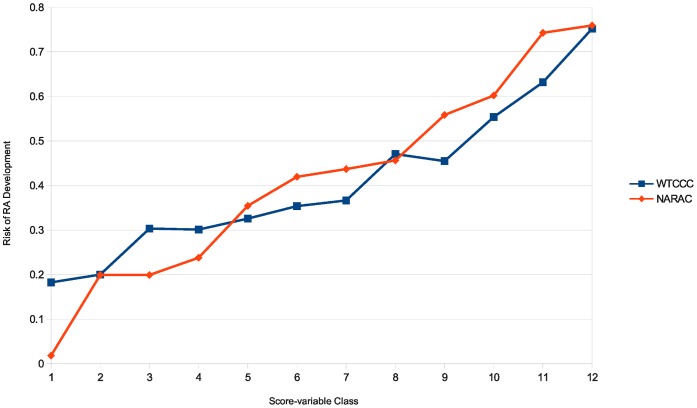
*Disease risk-Score class* diagram for RA *vs.* CTR and NARAC-A *vs.* NARAC-C comparisons. For each comparison overall score variable derived from the entire set of 44 SNPs present in the eight replicated RA-associated models was discretized into 12 bins, and for each bin the posterior probability of being affected by disease was calculated based on Bayes formula.

To illustrate how an increase in the value of the overall score variable influences the risk of disease development, the range of values corresponding to each overall score variable was discretized into 12 bins, and for each bin the posterior probability of being affected was calculated. As shown in Figure 2, for both case-control comparisons the risk of development of rheumatoid arthritis increases as the score takes larger values. Comparing RA *vs.* CTR, this risk ranges from around 18% when the score class is lowest to around 75% when the score class is highest. For model related to the NARAC-A *vs.* NARAC-C comparison’s model, the disease risk rises from around 2% for the lowest score class to around 75% for the highest score class. This indicates that the scores obtained from the total set of these 44 SNPs can be used as a predictor to estimate the probability by which an individual may develop the disease.

### Analysis of the Crohn’s Disease (CD) Dataset

**Figure 3:**
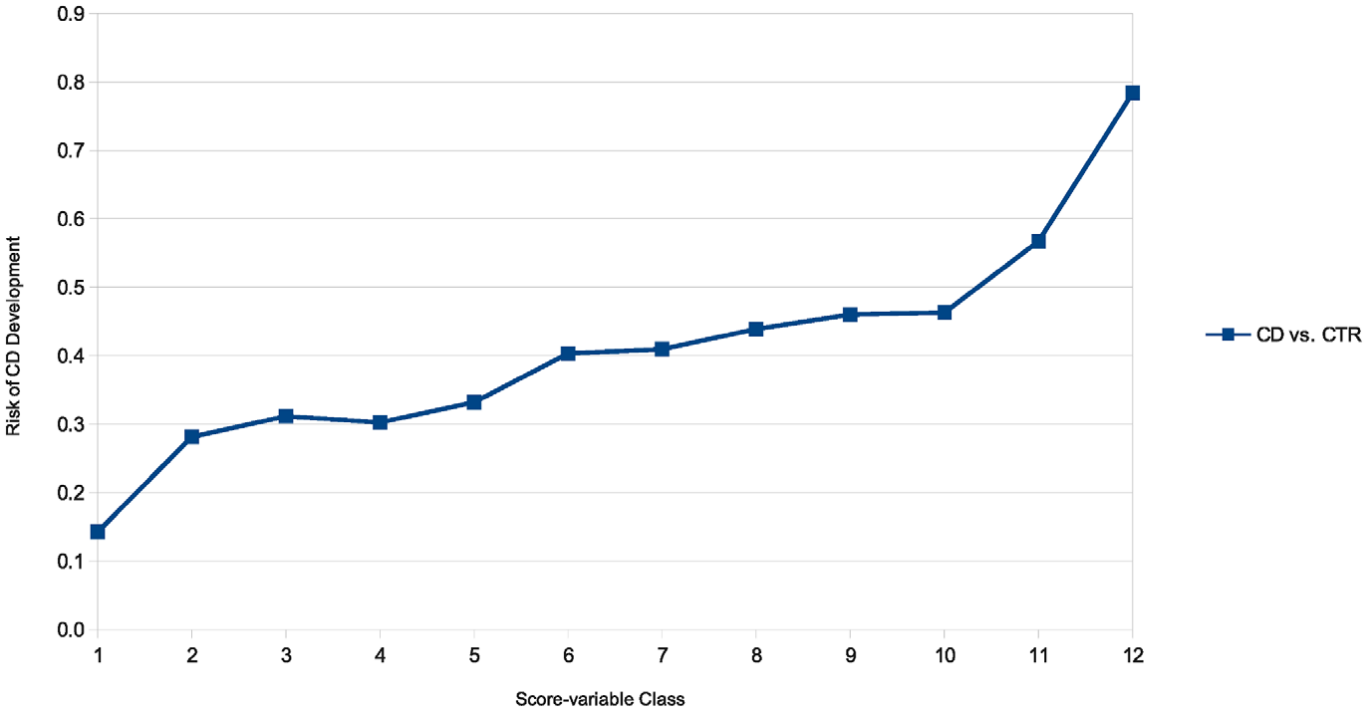
*Disease risk-Score class* diagram for CD *vs.* CTR. The overall score variable derived from the entire set of 57 SNPs present in the nine significant CD-associated models was discretized into 12 bins, and for each bin the posterior probability of being affected by disease was calculated based on Bayes formula.

The applicability of the KBAS method to other diseases was tested in Crohn’s disease (CD) using the same set of 24 pathways used for RA analysis. Detailed results are provided in Results S2. As seen in Tables S6, S7 and S8, successful models derived from nine pathways demonstrated evidence of association with Crohn’s disease. Eight out these nine pathways were also in association with RA in the WTCCC dataset, and six of them were also among the pathways replicated in the NARAC dataset. Two SNPs out of the 57 SNPs included in these models (see Table S9) are in linkage disequilibrium with two previously detected CD-associated SNPs. None of the other SNPs are among or in linkage disequilibrium with 71 known loci linked to CD previously [15]. The successful multi-SNP models included in the fitted regression model had odds ratios ranging from of 2.320 to 2.741 (see Table S10). Table S11 summarizes the results of a simple logistic regression analysis obtained by regressing disease state (CD *vs.* CTR) on the overall score variable derived from the entire set of 57 SNPs present in the nine CD-associated models. Table S12 provides a comparative summary of the observed disease-pathway associations analyzing RA and CD using WTCCC and NARAC datasets. Figure 3 illustrates the *Disease risk-Score class* diagram f*or* the CD vs. CTR comparison.

## Discussion

The method we presented in this paper provides a framework for extending genome-wide association studies to situations in which a phenotype is caused by a number of concurrent genetic factors. Its main purpose is to measure how well a multi-SNP model is able to classify case and control subjects for which genotype data are available. Biologically, multi-SNP models may implicate cases in which functional relationships exist among genetic factors of interest. These relationships might be for instance in the form of interaction among functionally redundant elements or physically interacting biomolecules, or of the concurrent occurrence of two or more hypomorphic mutations in different steps of a particular pathway [49], [50]. To capture these effects KBAS uses Genetic Algorithms, a powerful search method able to navigate through an extensive search space consisting of potential combinations of markers and to refine them by removing markers that show limited contribution to the trait under consideration. Instead of examining each marker individually, our method allows the user to define models consisting of a set of markers, to integrate the markers’ genotypes into a score variable and to evaluate the model’s ability to correctly classify cases and controls on the basis of the distribution of the score variable values.

Multi-SNP models that define a score variable over loci they contain are able to take into account the joint effects of loci individually conferring small risks to the phenotype under investigation, and the different combinations of trait influencing loci which is of considerable importance in capturing the genetic heterogeneity underlying a complex trait. For example we can imagine a case in which a hypothetical phenotype is produced by concurrent mutations in any two out of three loci involved in a particular pathway (e.g. loci A, B and C). Therefore, subjects having mutations in loci A and B show the same phenotype as subjects having mutations in loci A and C or loci B and C. This can be formally described using the following logical statement: [A *AND* B] *OR* [A *AND* C] *OR* [B and C]. However, since the main effect of each individual locus is small, subjects harboring single mutations do not develop the phenotype. Defining a score variable results in integrating small association signals arising from individual risk loci into an overall association signal capturing the joint effects of contributing loci. Moreover, since the score variable is computed independently on each subject, different combinations of loci can contribute to the same score variable distribution in parallel to each other, leading to a more accurate representation of the differences of case and control groups.

Since KBAS employs a Genetic Algorithm-based search engine, it enjoys high flexibility and efficiency in searching through an extremely large solution space. On the other hand, like with any other method relying on heuristic search algorithms, this does not ensure that the successful models are the absolute best ones. Our method is instead meant to be used in a hypothesis-based fashion: its purpose is not to discover the “best” set of markers that explains a phenotype; something that is computationally unfeasible since it would require testing all possible combinations of a given number of markers; but to confirm or invalidate the hypothesis that a user-provided set of markers is associated with the phenotype. The method does not require or stipulate a specific way of selecting markers: if the provided set does not contain a sufficient number of “relevant” ones, the algorithm will simply fail to find an appropriate combination of markers, and will report that the corresponding hypothesis is not supported by the available data.

This is clearly observed using the two sets of pathways we selected for this study, the first one containing pathways known to be relevant to the immune system, and the second one containing pathways unrelated to the immune system, used as negative controls. For instance, while on the first set of pathways KBAS was able to produce multiple replicated RA-associated models with remarkable classifying efficacy, none of the pathways in the second set gave rise to a successful model capable of significantly separating cases from controls (see Tables 2, 3 and 4).

In addition, our method provides researcher with a way to prioritize the successful models derived from different pathways in order to evaluate which pathway is more likely to be related to the pathogenesis of the disease of interest. For example comparing RA *vs.* CTR (see Table 4), the *complement and coagulation cascade* pathway seems to be of higher relevance to the process of development of rheumatoid arthritis compared to *T-cell receptor signaling* pathway. Also, among the significant models for the CD *vs.* CTR comparison (see Table S8), the *B-cell receptor signaling, cytokine-cytokine receptor interaction* and *T-cell receptor signaling* pathways show high association with the Crohn’s disease, while for example the association of the *antigen processing and presentation* pathway seems to be of lower importance compared to the three aforementioned pathways.

Another application of our method is to compare the contribution of specific pathways to different traits of interest. Although several pathways were labeled as associated with rheumatoid arthritis and Crohn’s disease in our analysis, each provides a different contribution to the diseases under investigation. For example comparing the two successful models derived from the *complement and coagulation cascade* pathway through the analysis of RA and CD datasets, RA-associated model has a smaller *p-*value than the CD-associated model, while the reverse is true for the two models derived from *T-cell receptor signaling* pathway (see Tables 4 and S8).

There are clear benefits to having SNPs selection be guided by preexisting biological knowledge. This choice maximizes the chance of including SNPs that are functionally related with the phenotype of interest, and makes it more likely that the results, if positive, may have an explicit biological interpretation. If the algorithm identifies a model with high case/control separation performance, it can be directly used to formulate a biological explanation for the observed phenotype. For example, this could involve identifying the genes containing the SNPs within the model or in LD with them, and making them high-priority candidates for further experimental analysis.

The observed significant associations between immune system related multi-SNP models and the diseases under investigation is consistent with previously revealed aspects of their pathogenesis as autoimmune diseases [1], [39], [43], [45]. The fact that a number of RA-associated models were replicated in another population of the same ancestry makes them a potential predictor which can be used to estimate the risk of disease development in individuals of Caucasian ancestry. As shown in the *Disease risk-Score class* diagrams, although the risk of disease development rises parallel to the increase in the score variable value, the disease risk in the lowest and highest classes, constituting extreme portions of the score-class spectrum, are not 0 or 100% respectively. This is consistent with the definition of complex diseases and indicates that there are still further genetic and non-genetic risk factors contributing to the RA and CD, which remain to be discovered.

Although we tested it using SNPs genotype data, KBAS can be applied to any kind of polymorphic genetic marker, provided that its alleles can be converted into numeric values in a way that consistently assigns values to different types of alleles (e.g. wild-type and mutant alleles). KBAS will then use the score variable obtained by combining these values to classify study subjects as affected or unaffected.

## Supporting Information

Table S1 **The number of genotyped and validated SNPs in each functional role class for the test and control pathways analyzed in this study.**
(DOC)

Table S2 **The list of SNPs included in the successful models showing strong or moderate association with rheumatoid arthritis. SNP positions refer to version GRCh37 of the human reference sequence.**
(DOC)

Table S3 **The list of SNPs from NARAC study replacing the SNPs from WTCCC study for replication of the results.**
(DOC)

Table S4 **Simple regression of disease-state on the overall score variable derived from the entire set of 44 SNPs present in the replicated RA-associated models (comparing RA** ***vs.*** **CTR).**
(DOC)

Table S5 **Simple regression of disease-state on the overall score variable derived from the entire set of 44 SNPs present in the replicated RA-associated models (comparing NARAC-A** ***vs.*** **NARAC-C).**
(DOC)

Table S6 **The** ***p-*** **values associated with the pairwise comparisons of the CD group and the two control groups using successful models derived from immune system related pathways.** The fitness *p-*values measure the fitness of each successful model retrieved by Genetic Algorithm engine. They are calculated by comparing original case and control datasets using corresponding successful models. Randomization-test *p-*values measure the significance of fitness *p-*values of their corresponding successful model by comparing permuted case and control datasets. According to Bonferroni’s correction, a fitness *p-*value <6.944×10^−9^ and a randomization test *p-*value <0.00104 were considered significant. The *p-*values of the models showing strong or moderate association with Crohn’s disease are in bold.
(DOC)

Table S7 **The** ***p-*** **values associated with the pairwise comparisons of the CD group and the two control groups using the successful models derived from negative control pathways.**
(DOC)

Table S8 **The** ***p-*** **values associated with the comparisons of the CD and CTR groups using successful models derived from pathways under consideration.** According to Bonferroni’s correction, a fitness *p-*value <6.944×10^−9^ and a randomization test *p-*value <0.00208 were considered significant. The *p -*values of models showing significant association with Crohn’s disease are in bold.
(DOC)

Table S9 **The list of SNPs included in the successful models showing association with Crohn’s disease.** SNP positions refer to version GRCh37 of the human reference sequence.
(DOC)

Table S10 **Multivariate regression of disease-state on the score variables derived from the successful models showing association with Crohn’s disease (comparing CD** ***vs.*** **CTR).**
(DOC)

Table S11 **Simple regression of disease-state on the overall score variable derived from the entire set of 57 SNPs present in the CD-associated models (comparing CD** ***vs.*** **CTR).**
(DOC)

Table S12 **Comparative summary of pathway associations with rheumatoid arthritis and Crohn’s disease.** (+) indicates that the successful model retrieved from the pathway is in strong association with the disease in the corresponding comparison, (−) indicates that the successful model retrieved from the pathway is not associated with the disease in the corresponding comparison, and (+/−) indicates that the successful model retrieved from the pathway is in borderline association with the disease in the corresponding comparison.
(DOC)

Methods S1 **Description of the method used to perform logistic regression analysis and to illustrate** ***Disease risk-Score class*** **diagram.**
(PDF)

Results S1 **Description of additional results regarding the analysis of rheumatoid arthritis (RA) dataset.**
(PDF)

Results S2 **Description of the results regarding the analysis of Crohn’s disease (CD) dataset.**
(PDF)
