# Supplementary material for: A Knowledge-Based Method for Association Studies on Complex Diseases
Source: PLoS One. 2012 Sep 6;7(9):e44162. doi: 10.1371/journal.pone.0044162 (PMC3435396; doi:10.1371/journal.pone.0044162)
Supplement: Table S1 — The number of genotyped and validated SNPs in each functional role class for the test and control pathways analyzed in this study. (DOC) [file pone.0044162.s001.doc]

Table S1: The number of genotyped and validated SNPs in each functional role class for the test and control pathways analyzed in this study.

| **Pathway** | **5'UTR** | **Promoter** | **Coding Sequence** | **Exon/Intron Boundary** | **3'UTR** | **Intron** | **Downstream** |
| --- | --- | --- | --- | --- | --- | --- | --- |
| **Test Pathways** | | | | | | | |
| **Antigen Processing and Presentation** | 1 | 22 | 6 | 18 | 10 | 76 | 15 |
| **B-cell Receptor Signaling** | 0 | 43 | 12 | 27 | 22 | 913 | 37 |
| **Chemokine Signaling** | 2 | 121 | 26 | 53 | 38 | 2213 | 123 |
| **Complement and Coagulation Cascades** | 0 | 47 | 27 | 43 | 22 | 500 | 34 |
| **Cytokine-Cytokine Receptor Interaction** | 4 | 168 | 37 | 67 | 57 | 1338 | 140 |
| **Fc Epsilon RI Signaling** | 1 | 58 | 18 | 29 | 19 | 1083 | 39 |
| **Fc Gamma R-mediated Phagocytosis** | 1 | 32 | 22 | 47 | 22 | 1668 | 50 |
| **Immune Network for IgA Production** | 0 | 12 | 0 | 8 | 8 | 92 | 12 |
| **Leukocyte Trans-endothelial Migration** | 5 | 74 | 27 | 33 | 23 | 1710 | 58 |
| **Natural Killer Cell Mediated Cytotoxicity** | 2 | 80 | 22 | 37 | 28 | 1038 | 55 |
| **Phagosome** | 0 | 54 | 20 | 62 | 19 | 822 | 52 |
| **Regulation of Autophagy** | 0 | 11 | 2 | 4 | 9 | 123 | 11 |
| **T-cell Receptor Signaling** | 1 | 74 | 15 | 34 | 28 | 1138 | 44 |
| **Toll-like Receptor Signaling** | 2 | 52 | 8 | 27 | 17 | 429 | 50 |
| **Control Pathways** | | | | | | | |
| **Cardiac Muscle Contraction** | 1 | 45 | 12 | 32 | 16 | 1298 | 26 |
| **Gap Junction** | 0 | 44 | 17 | 35 | 17 | 2095 | 49 |
| **Glycolysis/Gluconeogenesis** | 1 | 35 | 15 | 16 | 19 | 252 | 54 |
| **Insulin Signaling** | 0 | 61 | 26 | 42 | 30 | 1081 | 49 |
| **Nucleotide Excision Repair** | 0 | 22 | 9 | 35 | 8 | 226 | 19 |
| **Oxidative Phosphorylation** | 1 | 41 | 13 | 26 | 9 | 344 | 44 |
| **Purine Metabolism** | 2 | 76 | 22 | 79 | 33 | 2567 | 66 |
| **Pyrimidine Metabolism** | 1 | 35 | 11 | 39 | 22 | 585 | 31 |
| **Renin Angiotensin System** | 0 | 12 | 3 | 7 | 3 | 91 | 9 |
| **Spliceosome** | 2 | 38 | 7 | 40 | 27 | 294 | 66 |
