# Supplementary material for: A Knowledge-Based Method for Association Studies on Complex Diseases
Source: PLoS One. 2012 Sep 6;7(9):e44162. doi: 10.1371/journal.pone.0044162 (PMC3435396; doi:10.1371/journal.pone.0044162)
Supplement: Table S2 — The list of SNPs included in the successful models showing strong or moderate association with rheumatoid arthritis. SNP positions refer to version GRCh37 of the human reference sequence. (DOC) [file pone.0044162.s002.doc]

Table S2: The list of SNPs included in the successful models showing strong or moderate association with rheumatoid arthritis. SNP positions refer to version GRCh37 of the human reference sequence.

| **SNP** | **GENE** | | **NCBI GENE ID** | | | **CHROMOSOME** | | **POSITION** | | | | | **SNP-ROLE** |
| --- | --- | --- | --- | --- | --- | --- | --- | --- | --- | --- | --- | --- | --- |
| **Antigen Processing and Presentation Pathway** | | | | | | | | | | | | | |
| rs9272346 | | HLA-DQA1 | | 3117 | Chr6 | | | | 32,604,372 | | | Promoter | |
| rs7760860 | | NFYA | | 4800 | Chr6 | | | | 41,059,873 | | | Intron | |
| rs7869876 | | HSPA5 | | 3309 | Chr9 | | | | 127,994,564 | | | Downstream | |
| rs10163054 | | PDIA3 | | 2923 | Chr15 | | | | 44,047,638 | | | Intron | |
| **B-cell Receptor Signaling Pathway** | | | | | | | | | | | | | |
| rs2037547 | | GSK3B | | 2932 | Chr3 | | | | 119,544,615 | | | 3' UTR | |
| rs171649 | | PIK3R1 | | 5295 | Chr5 | | | | 67,569,746 | | | Exon/Intron boundary | |
| rs3735131 | | CARD11 | | 84433 | Chr7 | | | | 2,962,293 | | | Coding sequence | |
| rs2272733 | | IKBKB | | 3551 | Chr8 | | | | 42,157,902 | | | Intron | |
| rs4645869 | | FOS | | 2353 | Chr14 | | | | 75,749,875 | | | Downstream | |
| rs1013316 | | PRKCB | | 5579 | Chr16 | | | | 24,135,112 | | | Exon/Intron boundary | |
| rs2855259 | | BTK | | 695 | ChrX | | | | 100,615,478 | | | Exon/Intron boundary | |
| **Chemokine Signaling Pathway** | | | | | | | | | | | | | |
| rs17110519 | GNG5 | | | 2787 | | Chr1 | | | 84,968,749 | | Intron | | |
| rs2734871 | CXCR4 | | | 7852 | | Chr2 | | | 136,869,873 | | Downstream | | |
| rs5029748 | IKBKB | | | 3551 | | Chr8 | | | 42,140,549 | | Intron | | |
| rs3808391 | LYN | | | 4067 | | Chr8 | | | 56,790,649 | | Promoter | | |
| rs2839686 | CXCL12 | | | 6387 | | Chr10 | | | 44,881,455 | | Promoter | | |
| rs1950501 | ADCY4 | | | 196883 | | Chr14 | | | 24,806,800 | | Promoter | | |
| rs2075019 | SHC2 | | | 25759 | | Chr19 | | | 441,195 | | Intron | | |
| rs12394147 | PRKX | | | 5613 | | ChrX | | | 3,539,248 | | Exon/Intron boundary | | |
| **Complement and Coagulation Pathway** | | | | | | | | | | | | | |
| rs664142 | C8A | | | 731 | | Chr1 | | | 57,325,855 | | Intron | | |
| rs698094 | MASP1 | | | 5648 | | Chr3 | | | 186,970,808 | | Intron | | |
| rs2072633 | CFB | | | 629 | | Chr6 | | | 31,919,578 | | Intron | | |
| **Cytokine-Cytokine Receptor Interaction Pathway** | | | | | | | | | | | | | |
| rs3748669 | IL24 | | | 11009 | | Chr1 | | | 207,077,023 | | 3' UTR | | |
| rs11758366 | TNFRSF21 | | | 27242 | | Chr6 | | | 47,253,631 | | Exon/Intron boundary | | |
| rs334349 | TGFBR1 | | | 7046 | | Chr9 | | | 101,914,387 | | 3' UTR | | |
| rs2870946 | IL26 | | | 55801 | | Chr12 | | | 68,596,661 | | Intron | | |
| rs8107861 | IL29 | | | 282618 | | Chr19 | | | 39,784,152 | | Promoter | | |
| rs2834213 | IFNGR2 | | | 3460 | | Chr21 | | | 34,792,910 | | Intron | | |
| rs5980890 | EDA | | | 1896 | | ChrX | | | 69,232,272 | | Intron | | |
| **Fc Gamma R-mediated Phagocytosis Pathway** | | | | | | | | | | | | | |
| rs4971024 | PIP5K1A | | | 8394 | | | Chr1 | | 151,224,320 | Downstream | | | |
| rs10204214 | ASAP2 | | | 8853 | | | Chr2 | | 9,540,849 | Exon/Intron boundary | | | |
| rs352084 | MARCKS | | | 4082 | | | Chr6 | | 114,183,385 | 3' UTR | | | |
| rs7803810 | SCIN | | | 85477 | | | Chr7 | | 12,607,971 | Promoter | | | |
| rs12280627 | PAK1 | | | 5058 | | | Chr11 | | 77,047,679 | Intron | | | |
| rs901104 | GAB2 | | | 9846 | | | Chr11 | | 77,930,499 | Exon/Intron boundary | | | |
| rs307943 | PRKCG | | | 5582 | | | Chr19 | | 54,404,476 | Intron | | | |
| **Intestinal Immune Network for IgA Production Pathway** | | | | | | | | | | | | | |
| rs291092 | PIGR | | | 5284 | | | Chr1 | | 207,113,230 | Intron | | | |
| rs2734871 | CXCR4 | | | 7852 | | | Chr2 | | 136,869,873 | Downstream | | | |
| rs2236938 | CCR9 | | | 10803 | | | Chr3 | | 45,938,939 | Intron | | | |
| rs9272346 | HLA-DQA1 | | | 3117 | | | Chr6 | | 32,604,372 | Promoter | | | |
| rs1800795 | IL6 | | | 3569 | | | Chr7 | | 22,766,645 | Promoter | | | |
| rs11574530 | ITGB7 | | | 3695 | | | Chr12 | | 53,599,340 | Intron | | | |
| **Leukocyte Trans-endothelial Migration Pathway** | | | | | | | | | | | | | |
| rs6999346 | TRIM35 | | | 23087 | | | Chr8 | | 27,174,861 | Promoter | | | |
| rs2839686 | CXCL12 | | | 6387 | | | Chr10 | | 44,881,455 | Promoter | | | |
| rs16931177 | VCL | | | 7414 | | | Chr10 | | 75,870,890 | Intron | | | |
| rs11223714 | JAM3 | | | 83700 | | | Chr11 | | 134,016,011 | Exon/Intron boundary | | | |
| rs4802260 | VASP | | | 7408 | | | Chr19 | | 46,027,752 | Exon/Intron boundary | | | |
| rs219761 | CLDN14 | | | 23562 | | | Chr21 | | 37,839,410 | Promoter/Intron | | | |
| rs5921682 | NOX1 | | | 27035 | | | ChrX | | 100,130,437 | Promoter | | | |
| **Natural Killer Cell Mediated Cytotoxicity Pathway** | | | | | | | | | | | | | |
| rs164123 | SH2D1B | | | 117157 | | | Chr1 | | 162,367,654 | Intron | | | |
| rs1327473 | IFNGR1 | | | 3459 | | | Chr6 | | 137,541,230 | Promoter | | | |
| rs10234438 | RAC1 | | | 5879 | | | Chr7 | | 6,422,800 | Intron | | | |
| rs10111172 | TNFRSF10C | | | 8794 | | | Chr8 | | 22,969,091 | Intron | | | |
| rs16927724 | PRF1 | | | 5551 | | | Chr10 | | 72,363,568 | Promoter | | | |
| rs12597573 | NFATC3 | | | 4775 | | | Chr16 | | 68,167,521 | Intron | | | |
| rs11641233 | NFAT5 | | | 10725 | | | Chr16 | | 69,734,563 | 3' UTR | | | |
| rs307943 | PRKCG | | | 5582 | | | Chr19 | | 54,404,476 | Intron | | | |
| **Phagosome Pathway** | | | | | | | | | | | | | |
| rs17035245 | ATP6V1E2 | | | 90423 | | | Chr2 | | 46,739,683 | Coding sequence | | | |
| rs7622403 | ATP6V1A | | | 523 | | | Chr3 | | 113,472,434 | Intron | | | |
| rs1017813 | ITGB5 | | | 3693 | | | Chr3 | | 124,591,985 | Intron | | | |
| rs1032070 | ATP6V0A1 | | | 535 | | | Chr17 | | 40,618,251 | Intron | | | |
| rs5921682 | NOX1 | | | 27035 | | | ChrX | | 100,130,437 | Promoter | | | |
| **T-cell Receptor Signaling Pathway** | | | | | | | | | | | | | |
| rs27341 | ITK | | 3702 | | | Chr5 | | | 156,679,576 | Exon/Intron boundary | | | |
| rs3735131 | CARD11 | | 84433 | | | Chr7 | | | 2,962,293 | Coding sequence | | | |
| rs7358099 | CHUK | | 1147 | | | Chr10 | | | 101,955,002 | Intron | | | |
| rs152041 | CHP2 | | 63928 | | | Chr16 | | | 23,765,355 | Promoter | | | |
| rs3092923 | CD40L | | 959 | | | ChrX | | | 135,741,185 | Exon/Intron boundary | | | |
| **Toll-like Receptor Signaling Pathway** | | | | | | | | | | | | | |
| rs4256246 | CXCL10 | | 3627 | | | Chr4 | | | 76,945,522 | Promoter | | | |
| rs5743303 | TLR3 | | 7098 | | | Chr4 | | | 186,988,853 | Promoter | | | |
| rs3740720 | FADD | | 8772 | | | Chr11 | | | 70,053,547 | Downstream | | | |
| rs2403102 | TRAF3 | | 7187 | | | Chr14 | | | 103,306,215 | Intron | | | |
| rs3027898 | IRAK1 | | 3654 | | | ChrX | | | 153,275,890 | Downstream | | | |
