# Supplementary material for: A Knowledge-Based Method for Association Studies on Complex Diseases
Source: PLoS One. 2012 Sep 6;7(9):e44162. doi: 10.1371/journal.pone.0044162 (PMC3435396; doi:10.1371/journal.pone.0044162)
Supplement: Table S3 — The list of SNPs from NARAC study replacing the SNPs from WTCCC study for replication of the results. (DOC) [file pone.0044162.s003.doc]

Table S3: The list of SNPs from NARAC study replacing the SNPs from WTCCC study for replication of the results.

| **SNP in WTCCC Study** | **SNP in NARAC Study** | **Distance (bp)** | **SNP in WTCCC Study** | **SNP in NARAC Study** | **Distance (bp)** |
| --- | --- | --- | --- | --- | --- |
| rs10163054 | rs8040336 | 5,979 | rs1800795 | rs2069837 | 1,382 |
| rs9272346 | rs1063355 | 23,342 | rs291092 | rs291102 | 6,752 |
| rs7760860 | rs9296354 | 905 | rs11574530 | rs1554753 | 4,398 |
| rs7869876 | rs16927988 | 1,033 | rs6999346 | rs6986075 | 39 |
| rs17110519 | rs17365723 | 270 | rs16931177 | rs2279648 | 3,697 |
| rs2734871 | rs16832740 | 1,997 | rs11223714 | rs12803146 | 1,435 |
| rs2839686 | rs3780891 | 2,742 | rs4802260 | rs10995 | 2,402 |
| rs1950501 | rs3212247 | 2,995 | rs307943 | rs3745405 | 9,378 |
| rs2075019 | rs4919871 | 9,320 | rs1327473 | rs1327474 | 155 |
| rs664142 | rs669444 | 6,753 | rs10234438 | rs2347338 | 4,522 |
| rs7803810 | rs3735222 | 1,708 | rs11641233 | rs10517 | 9,197 |
| rs307943 | rs3745405 | 9,378 | rs12597573 | rs2418736 | 12,659 |
| rs352084 | rs7747961 | 7,049 | rs16927724 | rs3758562 | 1,488 |
| rs4971024 | rs7532935 | 13,531 | rs3735131 | rs1878805 | 879 |
| rs10204214 | rs10186062 | 1,437 | rs7358099 | rs12248110 | 246 |
| rs901104 | rs1318241 | 293 | rs152041 | rs194821 | 3,372 |
| rs12280627 | rs1377470 | 3,035 | rs3092923 | rs3092921 | 1,815 |
