# Supplementary material for: A Knowledge-Based Method for Association Studies on Complex Diseases
Source: PLoS One. 2012 Sep 6;7(9):e44162. doi: 10.1371/journal.pone.0044162 (PMC3435396; doi:10.1371/journal.pone.0044162)
Supplement: Table S4 — Simple regression of disease-state on the overall score variable derived from the entire set of 44 SNPs present in the replicated RA-associated models (comparing RA vs. CTR). (DOC) [file pone.0044162.s004.doc]

Table S4: Simple regression of disease-state on the overall score variable derived from the entire set of 44 SNPs present in the replicated RA-associated models (comparing RA *vs.* CTR).

| **Test of Overall Model** | | | | | | | | |
| --- | --- | --- | --- | --- | --- | --- | --- | --- |
| **Test** | | | **Chi-square** | **df** | ***P-*value** | | | |
| **Likelihood Ratio Test** | | | 391.2276 | 1 | <0.0001 | | | |
| **Score Test** | | | 368.9996 | 1 | <0.0001 | | | |
| **Wald Test** | | | 346.1146 | 1 | <0.0001 | | | |
| **Test of Parameters** | | | | | | | | |
| **Parameter** | **Parameter Estimate** | **Standard Error** | **Wald's Chi-square** | **df** | ***P-*value** | **Odds Ratio Estimates** | | |
|  |  |  |  |  |  | **Point Estimate** | **95% Confidence Interval** | |
| **Intercept** | -0.4095 | 0.0301 | 185.4796 | 1 | <0.0001 | - | - | - |
| **Score** | 0.8745 | 0.0470 | 346.1146 | 1 | <0.0001 | 2.398 | 2.187 | 2.629 |
| **Goodness-of-fit Test** | | | | | | | | |
| **Test** | | | **Chi-square** | **df** | ***P-*value** | | | |
| **Hosmer - Lemeshow Test** | | | 9.2494 | 8 | 0.3217 | | | |
