# Supplementary material for: A Knowledge-Based Method for Association Studies on Complex Diseases
Source: PLoS One. 2012 Sep 6;7(9):e44162. doi: 10.1371/journal.pone.0044162 (PMC3435396; doi:10.1371/journal.pone.0044162)
Supplement: Table S5 — Simple regression of disease-state on the overall score variable derived from the entire set of 44 SNPs present in the replicated RA-associated models (comparing NARAC-A vs. NARAC-C). (DOC) [file pone.0044162.s005.doc]

Table S5: Simple regression of disease-state on the overall score variable derived from the entire set of 44 SNPs present in the replicated RA-associated models (comparing NARAC-A *vs.* NARAC-C).

| **Test of Overall Model** | | | | | | | | |
| --- | --- | --- | --- | --- | --- | --- | --- | --- |
| **Test** | | | **Chi-square** | **df** | ***P-*value** | | | |
| **Likelihood Ratio Test** | | | 325.0076 | 1 | < 0.0001 | | | |
| **Score Test** | | | 295.5573 | 1 | < 0.0001 | | | |
| **Wald Test** | | | 259.0395 | 1 | < 0.0001 | | | |
| **Test of Parameters** | | | | | | | | |
| **Parameter** | **Parameter Estimate** | **Standard Error** | **Wald's Chi-square** | **df** | ***P-*value** | **Odds Ratio Estimates** | | |
|  |  |  |  |  |  | **Point Estimate** | **95% Confidence Interval** | |
| **Intercept** | 0.2371 | 0.0561 | 17.8931 | 1 | <0.0001 | - | - | - |
| **Score** | 1.0151 | 0.0631 | 259.0395 | 1 | <0.0001 | 2.760 | 2.439 | 3.123 |
| **Goodness-of-fit Test** | | | | | | | | |
| **Test** | | | **Chi-square** | **df** | ***P-*value** | | | |
| **Hosmer - Lemeshow Test** | | | 9.5291 | 8 | 0.2996 | | | |
