# Supplementary material for: A Knowledge-Based Method for Association Studies on Complex Diseases
Source: PLoS One. 2012 Sep 6;7(9):e44162. doi: 10.1371/journal.pone.0044162 (PMC3435396; doi:10.1371/journal.pone.0044162)
Supplement: Table S6 — The p- values associated with the pairwise comparisons of the CD group and the two control groups using successful models derived from immune system related pathways. The fitness p-values measure the fitness of each successful model retrieved by Genetic Algorithm engine. They are calculated by comparing original case and control datasets using corresponding successful models. Randomization-test p-values measure the significance of fitness p-values of their corresponding successful model by comparing permuted case and control datasets. According to Bonferroni’s correction, a fitness p-value <6.944×10^−9^ and a randomization test p-value <0.00104 were considered significant. The p-values of the models showing strong or moderate association with Crohn’s disease are in bold. (DOC) [file pone.0044162.s006.doc]

Table S6: The *p-*values associated with the pairwise comparisons of the CD group and the two control groups using the successful models derived from immune system related pathways. The fitness *p-*values measure the fitness of each successful model retrieved by Genetic Algorithm engine. They are calculated by comparing original case and control datasets using corresponding successful models. Randomization-test *p-*values measure the significance of fitness *p-*values of their corresponding successful model by comparing permuted case and control datasets. According to Bonferroni's correction, a fitness *p-*value < 6.944x10^-9^ and a randomization test *p-*value < 0.00104 were considered significant. The *p-*values of the models showing strong or moderate association with Crohn's disease are in bold.

| **Pathway** | **58C *vs.* NBS** | **CD *vs.* 58C** | | **CD *vs.* NBS** | |
| --- | --- | --- | --- | --- | --- |
|  | **Fitness** | **Fitness** | **Randomization-test** | **Fitness** | **Randomization-test** |
| **Antigen Processing and Presentation** | > 0.05 | 2.36x10^-6^ | 0.00605 | 7.85x10^-6^ | 0.01423 |
| **B-cell Receptor Signaling** | > 0.05 | **1.07x10^-10^** | **0.00018** | **5.01x10^-11^** | **0.00002** |
| **Chemokine Signaling** | > 0.05 | 0.00015 | 0.04892 | 0.00085 | > 0.05 |
| **Complement and Coagulation Cascades** | > 0.05 | 1.44x10^-6^ | 0.02870 | 6.81x10^-6^ | > 0.05 |
| **Cytokine-Cytokine Receptor Interaction** | 0.04556 | **7.01x10^-11^** | **0.00121** | **3.22x10^-10^** | **0.00367** |
| **Fc Epsilon RI Signaling** | > 0.05 | 0.00877 | > 0.05 | 0.00529 | > 0.05 |
| **Fc Gamma R-mediated Phagocytosis** | > 0.05 | > 0.05 | > 0.05 | > 0.05 | > 0.05 |
| **Immune Network for IgA Production** | > 0.05 | 4.51x10^-7^ | 0.00304 | 6.03x10^-6^ | 0.02240 |
| **Leukocyte Trans-endothelial Migration** | > 0.05 | 1.21x10^-6^ | > 0.05 | 4.89x10^-7^ | 0.03103 |
| **Natural Killer Cell Mediated Cytotoxicity** | > 0.05 | 2.24x10^-5^ | 0.03038 | 2.58x10^-6^ | 0.00620 |
| **Phagosome** | > 0.05 | 0.00021 | > 0.05 | 4.61x10^-5^ | > 0.05 |
| **Regulation of Autophagy** | > 0.05 | 9.94x10^-5^ | > 0.05 | 0.00031 | > 0.05 |
| **T-cell Receptor Signaling** | 0.00783 | **1.40x10^-11^** | **0.00002** | **8.30x10^-11^** | **0.00015** |
| **Toll-like Receptor Signaling** | > 0.05 | 0.00113 | 0.02412 | 0.00511 | > 0.05 |
