# Supplementary material for: A Knowledge-Based Method for Association Studies on Complex Diseases
Source: PLoS One. 2012 Sep 6;7(9):e44162. doi: 10.1371/journal.pone.0044162 (PMC3435396; doi:10.1371/journal.pone.0044162)
Supplement: Table S7 — The p- values associated with the pairwise comparisons of the CD group and the two control groups using the successful models derived from negative control pathways. (DOC) [file pone.0044162.s007.doc]

Table S7: The *p-*values associated with the pairwise comparisons of the CD group and the two control groups using the successful models derived from negative control pathways.

| **Pathway** | **58C *vs.* NBS** | **CD *vs.* 58C** | | **CD *vs.* NBS** | |
| --- | --- | --- | --- | --- | --- |
|  | **Fitness** | **Fitness** | **Randomization-test** | **Fitness** | **Randomization-test** |
| **Cardiac Muscle Contraction** | > 0.05 | 5.64x10^-6^ | > 0.05 | 3.43x10^-5^ | > 0.05 |
| **Gap Junction** | > 0.05 | 6.42x10^-6^ | > 0.05 | 6.88x10^-6^ | > 0.05 |
| **Glycolysis/Gluconeogenesis** | > 0.05 | 0.00502 | > 0.05 | 0.00691 | > 0.05 |
| **Insulin Signaling** | 0.02463 | 7.86x10^-6^ | > 0.05 | 9.54x10^-7^ | > 0.05 |
| **Nucleotide Excision Repair** | > 0.05 | 0.00738 | > 0.05 | 0.00482 | > 0.05 |
| **Oxidative Phosphorylation** | > 0.05 | 9.84x10^-7^ | 0.02836 | 5.25x10^-7^ | 0.01937 |
| **Purine Metabolism** | > 0.05 | 0.00017 | > 0.05 | 1.40x10^-6^ | 0.03845 |
| **Pyrimidine Metabolism** | > 0.05 | 0.00069 | > 0.05 | 1.41x10^-5^ | > 0.05 |
| **Renin Angiotensin System** | > 0.05 | 0.00028 | > 0.05 | 0.00105 | > 0.05 |
| **Spliceosome** | > 0.05 | 1.13x10^-6^ | > 0.05 | 5.71x10^-6^ | > 0.05 |
