# Supplementary material for: A Knowledge-Based Method for Association Studies on Complex Diseases
Source: PLoS One. 2012 Sep 6;7(9):e44162. doi: 10.1371/journal.pone.0044162 (PMC3435396; doi:10.1371/journal.pone.0044162)
Supplement: Table S8 — The p- values associated with the comparisons of the CD and CTR groups using successful models derived from pathways under consideration. According to Bonferroni’s correction, a fitness p-value <6.944×10^−9^ and a randomization test p-value <0.00208 were considered significant. The p -values of models showing significant association with Crohn’s disease are in bold. (DOC) [file pone.0044162.s008.doc]

Table S8: The *p-*values associated with the comparisons of the CD and CTR groups using the successful models derived from pathways under consideration. According to Bonferroni's correction, a fitness *p-*value < 6.944x10^-9^ and a randomization test *p-*value < 0.00208 were considered significant. The *p -*values of models showing significant association with Crohn's disease are in bold.

| **Pathway** | **Fitness** | **Randomization-test** |
| --- | --- | --- |
| **Test Pathways** | | |
| **Antigen Processing and Presentation** | **4.50x10 ^-9^** | **0.00003** |
| **B-cell Receptor Signaling** | **4.01x10 ^-15^** | **< 10^-5^** |
| **Chemokine Signaling** | 8.59x10 ^-6^ | 0.00523 |
| **Complement and Coagulation Cascades** | **4.32x10 ^-9^** | **0.00053** |
| **Cytokine-Cytokine Receptor Interaction** | **1.16x10 ^-15^** | **< 10^-5^** |
| **Fc Epsilon RI Signaling** | 0.00204 | 0.03899 |
| **Fc Gamma R-mediated Phagocytosis** | > 0.05 | > 0.05 |
| **Intestinal Immune Network for IgA Production** | **1.76x10 ^-9^** | **0.00005** |
| **Leukocyte Trans-endothelial Migration** | **1.49x10 ^-9^** | **0.00072** |
| **Natural Killer Cell Mediated Cytotoxicity** | **7.40x10 ^-9^** | **0.00005** |
| **Phagosome** | 2.75x10 ^-6^ | > 0.05 |
| **Regulation of Autophagy** | 3.51x10 ^-6^ | 0.01377 |
| **T-cell Receptor Signaling** | **6.51x0 ^-15^** | **< 10^-5^** |
| **Toll-like Receptor Signaling** | 8.61x10 ^-5^ | 0.00240 |
| **Control Pathways** | | |
| **Cardiac Muscle Contraction** | 2.36x10 ^-7^ | 0.01646 |
| **Gap Junction** | 1.01x10 ^-7^ | 0.00479 |
| **Glycolysis/ Gluconeogenesis** | 0.00207 | > 0.05 |
| **Insulin Signaling** | 1.15x10 ^-8^ | 0.01003 |
| **Nucleotide Excision Repair** | 0.00109 | > 0.05 |
| **Oxidative Phosphorylation** | **3.57x10 ^-9^** | **0.00085** |
| **Purine Metabolism** | 8.92x10 ^-7^ | 0.02956 |
| **Pyrimidine Metabolism** | 9.19x10 ^-6^ | > 0.05 |
| **Renin Angiotensin System** | 3.19x10 ^-5^ | 0.04161 |
| **Spliceosome** | 1.42x10 ^-8^ | 0.00359 |
