# Supplementary material for: A Knowledge-Based Method for Association Studies on Complex Diseases
Source: PLoS One. 2012 Sep 6;7(9):e44162. doi: 10.1371/journal.pone.0044162 (PMC3435396; doi:10.1371/journal.pone.0044162)
Supplement: Table S9 — The list of SNPs included in the successful models showing association with Crohn’s disease. SNP positions refer to version GRCh37 of the human reference sequence. (DOC) [file pone.0044162.s009.doc]

Table S9: The list of SNPs included in the successful models showing association with Crohn's disease. SNP positions refer to version GRCh37 of the human reference sequence.

| **SNP** | | | **GENE** | | | | **GENE IDENTIFIER** | **CHROMOSOME** | | | **POSITION** | | **SNP-ROLE** |
| --- | --- | --- | --- | --- | --- | --- | --- | --- | --- | --- | --- | --- | --- |
| **Antigen Processing and Presentation Pathway** | | | | | | | | | | | | | |
| rs2569094 | | | CD74 | | | | 972 | Chr5 | | | 149,795,052 | | Promoter |
| rs504697 | | | HSP90AB1 | | | | 3326 | Chr6 | | | 44,220,076 | | Exon/Intron boundary |
| rs7869876 | | | HSPA5 | | | | 3309 | Chr9 | | | 127,994,564 | | Downstream |
| rs10163054 | | | PDIA3 | | | | 2923 | Chr15 | | | 44,047,638 | | Intron |
| **B-cell Receptor Signaling Pathway** | | | | | | | | | | | | | |
| rs9724615 | | NRAS | | | 4893 | | | | Chr1 | | 115,259,550 | | Promoter |
| rs2037547 | | GSK3B | | | 2932 | | | | Chr3 | | 119,544,615 | | 3' UTR |
| rs767652 | | DAPP1 | | | 27071 | | | | Chr4 | | 100,791,805 | | Downstream |
| rs2272733 | | IKBKB | | | 3551 | | | | Chr8 | | 42,157,902 | | Intron |
| rs290990 | | SYK | | | 6850 | | | | Chr9 | | 93,561,695 | | Promoter |
| rs6096473 | | NFATC2 | | | 4773 | | | | Chr20 | | 50,181,272 | | Promoter |
| rs2855259 | | BTK | | | 695 | | | | ChrX | | 100,615,478 | | Exon/Intron boundary |
| **Complement and Coagulation Cascades** | | | | | | | | | | | | | |
| rs2935542 | | C1QA | | | 712 | | | | Chr1 | | 22,963,471 | | Intron |
| rs671137 | | C8A | | | 731 | | | | Chr1 | | 57,319,480 | | Promoter |
| rs428060 | | CFH | | | 3075 | | | | Chr1 | | 196,706,200 | | Exon/Intron boundary |
| rs11117913 | | CR2 | | | 1380 | | | | Chr1 | | 207,668,235 | | Downstream |
| rs7096206 | | MBL2 | | | 4153 | | | | Chr10 | | 54,531,685 | | Promoter |
| rs3213721 | | VWF | | | 7450 | | | | Chr12 | | 6,182,753 | | Exon/Intron boundary |
| rs5960 | | F10 | | | 2159 | | | | Chr13 | | 113,801,737 | | Coding sequence |
| **Cytokine-Cytokine Receptor Interaction Pathway** | | | | | | | | | | | | | |
| rs3748669 | | IL24 | | 11009 | | | | Chr1 | | 207,077,023 | | | 3' UTR |
| rs2192752 | | IL1R1 | | 3554 | | | | Chr2 | | 102,769,373 | | | Promoter |
| rs2270418 | | TNFSF10 | | 8743 | | | | Chr3 | | 172,240,999 | | | Exon/Intron boundary |
| rs13143866 | | IL21 | | 59067 | | | | Chr4 | | 123,540,758 | | | Intron |
| rs6876446 | | LIFR | | 3977 | | | | Chr5 | | 38,474,149 | | | Downstream |
| rs1327473 | | IFNGR1 | | 3459 | | | | Chr6 | | 137,541,230 | | | Promoter |
| rs1800795 | | IL6 | | 3569 | | | | Chr7 | | 22,766,645 | | | Promoter |
| rs6557634 | | TNFRSF10A | | 8797 | | | | Chr8 | | 23,060,256 | | | Coding sequence |
| rs11818239 | | BMPR1A | | 657 | | | | Chr10 | | 88,659,788 | | | Coding sequence |
| rs784894 | | AMHR2 | | 269 | | | | Chr12 | | 53,817,956 | | | Intron |
| rs11104905 | | KITLG | | 4254 | | | | Chr12 | | 88,890,581 | | | 3' UTR |
| **Intestinal Immune Network for IgA Production Pathway** | | | | | | | | | | | | | |
| rs1554286 | IL10 | | | | | 3586 | | Chr1 | | | 206,944,233 | Exon/Intron boundary | |
| rs6767853 | CD80 | | | | | 941 | | Chr3 | | | 119,252,238 | Intron | |
| rs2715260 | CD86 | | | | | 942 | | Chr3 | | | 121,793,490 | Intron | |
| rs6886399 | CCL28 | | | | | 56477 | | Chr5 | | | 43,413,268 | Promoter | |
| rs1800795 | IL6 | | | | | 3569 | | Chr7 | | | 22,766,645 | Promoter | |
| rs11574530 | ITGB7 | | | | | 3695 | | Chr12 | | | 53,599,340 | Intron | |

Table S9 (continue).

| **Leukocyte Trans-endothelial Migration Pathway** | | | | | | | |
| --- | --- | --- | --- | --- | --- | --- | --- |
| rs497900 | PIK3CB | 5291 | Chr3 | | 138,433,568 | | Exon/Intron boundary |
| rs11966646 | CLDN20 | 49861 | Chr6 | | 155,585,154 | | 5' UTR |
| rs2839686 | CXCL12 | 6387 | Chr10 | | 44,881,455 | | Promoter |
| rs3802903 | ESAM | 90952 | Chr11 | | 124,634,946 | | Promoter |
| rs568259 | VAV1 | 7409 | Chr19 | | 6,782,574 | | Intron |
| rs4802260 | VASP | 7408 | Chr19 | | 46,027,752 | | Exon/Intron boundary |
| rs219761 | CLDN14 | 23562 | Chr21 | | 37,839,410 | | Intron |
| **Natural killer Cell Mediated Cytotoxicity Pathway** | | | | | | | |
| rs13112866 | PPP3CA | 5530 | Chr4 | | 102,120,454 | | Intron |
| rs1327473 | IFNGR1 | 3459 | Chr6 | | 137,541,230 | | Promoter |
| rs2126053 | SYK | 6850 | Chr9 | | 93,650,015 | | Exon/Intron boundary |
| rs1062124 | SHC4 | 399694 | Chr15 | | 49,117,549 | | 3' UTR |
| **T-cell Receptor Signaling Pathway** | | | | | | | |
| rs7555443 | PTPRC | 5788 | Chr1 | | 198,707,217 | | Intron |
| rs1554286 | IL10 | 3586 | Chr1 | | 206,944,233 | | Exon/Intron boundary |
| rs7582886 | NCK2 | 8440 | Chr2 | | 106,359,698 | | Promoter |
| rs6784820 | RHOA | 387 | Chr3 | | 49,450,864 | | Promoter |
| rs3808917 | CHUK | 1147 | Chr10 | | 101,990,715 | | Promoter |
| rs12599391 | NFAT5 | 10725 | Chr16 | | 69,605,349 | | Intron |
| rs8090560 | NFATC1 | 4772 | Chr18 | | 77,213,155 | | Intron |
| **Oxidative Phosphorylation Pathway** | | | | | | | |
| rs2726502 | PPA2 | 27068 | | Chr4 | 106,298,896 | Intron | |
| rs2029623 | ATP6V1F | 9296 | | Chr7 | 128,507,297 | Downstream | |
| rs2244916 | PPA1 | 5464 | | Chr10 | 71,969,133 | Exon/Intron boundary | |
| rs1000984 | COX15 | 1355 | | Chr10 | 101,470,085 | 3' UTR | |
| rs3794186 | TCIRG1 | 10312 | | Chr11 | 67,821,036 | Downstream | |
| rs9630712 | COX10 | 1352 | | Chr17 | 13,970,617 | Promoter | |
| rs1032070 | ATP6V0A1 | 535 | | Chr17 | 40,618,251 | Intron | |
