# Supplementary material for: A Knowledge-Based Method for Association Studies on Complex Diseases
Source: PLoS One. 2012 Sep 6;7(9):e44162. doi: 10.1371/journal.pone.0044162 (PMC3435396; doi:10.1371/journal.pone.0044162)
Supplement: Table S10 — Multivariate regression of disease-state on the score variables derived from the successful models showing association with Crohn’s disease (comparing CD vs. CTR). (DOC) [file pone.0044162.s010.doc]

Table S10: Multivariate regression of disease-state on the score variables derived from the successful models showing association with Crohn's disease (comparing CD *vs.* CTR).

| **Test of Overall Model** | | | | | | | | |
| --- | --- | --- | --- | --- | --- | --- | --- | --- |
| **Test** | | | **Chi-square** | **df** | ***P-*value** | | | |
| **Likelihood Ratio Test** | | | 259.5042 | 7 | <0.0001 | | | |
| **Score Test** | | | 194.4606 | 7 | <0.0001 | | | |
| **Wald Test** | | | 214.6265 | 7 | <0.0001 | | | |
| **Test of Parameters** | | | | | | | | |
| **Parameter** | **Parameter Estimate** | **Standard Error** | **Wald's Chi-square** | **df** | ***P-*value** | **Odds Ratio Estimates** | | |
|  |  |  |  |  |  | **Point Estimate** | **95% Confidence Interval** | |
| **Intercept** | -0.3866 | 0.0296 | 170.0454 | 1 | <0.0001 | - | - | - |
| **Pathway 1** | 0.9332 | 0.2290 | 16.6024 | 1 | 0.0001 | 2.543 | 1.623 | 3.983 |
| **Pathway 2** | 0.9589 | 0.1375 | 48.6488 | 1 | <0.0001 | 2.609 | 1.993 | 3.416 |
| **Pathway 3** | 0.8805 | 0.1871 | 22.1391 | 1 | <0.0001 | 2.412 | 1.671 | 3.481 |
| **Pathway 4** | 0.9304 | 0.1454 | 40.9663 | 1 | <0.0001 | 2.535 | 1.907 | 3.371 |
| **Pathway 5** | 1.0084 | 0.2125 | 22.5234 | 1 | <0.0001 | 2.741 | 1.808 | 4.157 |
| **Pathway6** | 0.9256 | 0.1392 | 44.2113 | 1 | <0.0001 | 2.523 | 1.921 | 3.315 |
| **Pathway 7** | 0.8416 | 0.1852 | 20.6553 | 1 | <0.0001 | 2.320 | 1.614 | 3.335 |
| **Goodness-of-fit Test** | | | | | | | | |
| **Test** | | | **Chi-square** | **df** | ***P-*value** | | | |
| **Hosmer - Lemeshow Test** | | | 7.7630 | 8 | 0.4570 | | | |

Pathway 1: Antigen Processing and Presentation Pathway

Pathway 2: B-cell Receptor Signaling Pathway

Pathway 3: Complement and Coagulation Cascades Pathway

Pathway 4: Cytokine -Cytokine Receptor Interaction Pathway

Pathway 5: Leukocyte Trans-endothelial Migration Pathway

Pathway 6: T-cell Receptor Signaling Pathway

Pathway 7: Oxidative Phosphorylation Pathway
