# Supplementary material for: A Knowledge-Based Method for Association Studies on Complex Diseases
Source: PLoS One. 2012 Sep 6;7(9):e44162. doi: 10.1371/journal.pone.0044162 (PMC3435396; doi:10.1371/journal.pone.0044162)
Supplement: Table S12 — Comparative summary of pathway associations with rheumatoid arthritis and Crohn’s disease. (+) indicates that the successful model retrieved from the pathway is in strong association with the disease in the corresponding comparison, (−) indicates that the successful model retrieved from the pathway is not associated with the disease in the corresponding comparison, and (+/−) indicates that the successful model retrieved from the pathway is in borderline association with the disease in the corresponding comparison. (DOC) [file pone.0044162.s012.doc]

Table S12: Comparative summary of pathway associations with rheumatoid arthritis and Crohn's disease. (+) indicates that the successful model retrieved from the pathway is in strong association with the disease in the corresponding comparison, (-) indicates that the successful model retrieved from the pathway is not associated with the disease in the corresponding comparison, and (+/-) indicates that the successful model retrieved from the pathway is in borderline association with the disease in the corresponding comparison.

| **Pathway** | **RA *vs.* CTR** | **NARAC-A *vs.* NARAC-C** | **CD *vs.* CTR** |
| --- | --- | --- | --- |
| **Test Pathways** |  |  |  |
| **Antigen Processing and Presentation** | **+** | **+** | **+** |
| **B-cell Receptor Signaling** | **+** | - | **+** |
| **Chemokine Signaling** | **+** | +/- | - |
| **Complement and Coagulation Cascades** | **+** | **+** | **+** |
| **Cytokine-Cytokine Receptor Interaction** | **+** | - | **+** |
| **Fc Epsilon RI Signaling** | - | - | - |
| **Fc Gamma R-mediated Phagocytosis** | **+** | **+** | - |
| **Intestinal Immune Network for IgA Production** | **+** | **+** | **+** |
| **Leukocyte Trans-endothelial Migration** | **+** | +/- | **+** |
| **Natural Killer Cell Mediated Cytotoxicity** | **+** | +/- | **+** |
| **Phagosome** | **+** | - | - |
| **Regulation of Autophagy** | - | - | - |
| **T-cell Receptor Signaling** | **+** | **+** | **+** |
| **Toll-like Receptor Signaling** | **+** | - | - |
| **Control Pathways** |  |  |  |
| **Cardiac Muscle Contraction** | **-** | **-** | **-** |
| **Gap Junction** | **-** | **-** | **-** |
| **Glycolysis/Gluconeogenesis** | **-** | **-** | **-** |
| **Insulin Signaling** | **-** | **-** | **-** |
| **Nucleotide Excision Repair** | **-** | **-** | **-** |
| **Oxidative Phosphorylation** | **-** | **-** | **+** |
| **Purine Metabolism** | **-** | **-** | **-** |
| **Pyrimidine Metabolism** | **-** | **-** | **-** |
| **Renin Angiotensin System** | **-** | **-** | **-** |
| **Spliceosome** | **-** | **-** | **-** |
