# Supplementary material for: A Knowledge-Based Method for Association Studies on Complex Diseases
Source: PLoS One. 2012 Sep 6;7(9):e44162. doi: 10.1371/journal.pone.0044162 (PMC3435396; doi:10.1371/journal.pone.0044162)
Supplement: Methods S1 — Description of the method used to perform logistic regression analysis and to illustrate Disease risk-Score class diagram. (PDF) [file pone.0044162.s013.pdf]

A Knowledge-based Method for Association Studies on Complex Diseases.

Alireza Nazarian, Heike Sichtig, Alberto Riva

Department of Molecular Genetics and Microbiology & UF Genetics Institute, University of Florida, Gainesville, FL, USA

Supplementary Materials – Methods S1

Logistic Regression Analysis and Disease Risk-Score Class Diagram

For each subject in the case and control groups, an overall score variable is computed on the basis of the entire set of SNPs selected by the disease-associated successful models. We then fit a simple logistic regression model using the overall scores as the independent variable and the disease state as the response variable. The purpose of this is to evaluate the applicability of the entire set of SNPs present in disease-associated models in predicting the risk of the disease.

The overall score variable is then discretized into multiple quantile classes and a *Disease risk-Score class diagram* is illustrated by computing the posterior probability of disease development for each class using Bayes' formula [1]:

$$P(affected \mid sc) = \frac{P(sc \mid affected) * P(affected)}{P(sc \mid affected) * P(affected) + P(sc \mid healthy) * P(healthy)}$$

where *sc* is the score class, and P(*affected*) and P(*healthy*) are the prior probabilities of a subject being in the affected or healthy groups, respectively. This procedure was used to generate Figures 2 and 3 in the main text.

References

1. Bolstad WM (2007) Introduction to Bayesian Statistics. 2nd ed. Wiley-Interscience. 464 p.
