# Supplementary material for: A Knowledge-Based Method for Association Studies on Complex Diseases
Source: PLoS One. 2012 Sep 6;7(9):e44162. doi: 10.1371/journal.pone.0044162 (PMC3435396; doi:10.1371/journal.pone.0044162)
Supplement: Results S1 — Description of additional results regarding the analysis of rheumatoid arthritis (RA) dataset. (PDF) [file pone.0044162.s014.pdf]

A Knowledge-based Method for Association Studies on Complex Diseases.

Alireza Nazarian, Heike Sichtig, Alberto Riva

Department of Molecular Genetics and Microbiology & UF Genetics Institute, University of Florida, Gainesville, FL, USA

Supplementary Materials – Results S1

Analysis of the Rheumatoid Arthritis (RA) Dataset

RA *vs.* CTR

To further investigate the goodness-of-fit of the derived successful models, we compared the case group with the pooled population of the two control groups present in the WTCCC dataset [1], here called CTR. The score variable was generated using the same procedure as in the previous case-control comparisons (i.e. RA *vs.* NBS and RA *vs.* 58C) and the statistical significance of the fitness *p*-value resulting from each model was validated through permutation testing. The significance threshold for interpreting fitness *p*-values is the same as the one used in previous step. Given the total number of pairwise comparisons (n=24), an adjusted significance level of 0.00208 was applied to interpret the results of permutation test according to Bonferroni's correction [2]. A model was considered significant if both its fitness and randomization test *p*-values were significant.

As shown in Table 4, all the eleven models found to be in strong or moderate association with RA in the previous step (RA *vs.* NBS and RA *vs.* 58C) showed statistically significant performance in separating RA from CTR, with fitness values less than 2.90×10^{-10} which were then validated by permutation test *p*-values less than 2×10^{-5}. In addition, the model from the *Fc gamma R-mediated phagocytosis* pathway also yielded significant fitness and permutation test *p*-values ($p < 9.09 \times 10^{-10}$ and $p < 3 \times 10^{-4}$ respectively). Although the multi-SNP model related to the *oxidative phosphorylation* pathway had a significant fitness *p*-value ($p < 1.72 \times 10^{-9}$), its corresponding permutation test *p*-value was not significant ($p = 0.0046$).

Simple Logistic Regression Analysis

The disease state was regressed on the overall score variable computed based on the all 44 SNPs present in the eight models replicated in the NARAC dataset [3] (see Table 4 and Table S2). The fitted regression models for RA *vs.* CTR and NARAC-A *vs.* NARAC-C comparisons resulted in overall model *p*-values less than 10^{-4}, and the covariates of the overall score variables were statistically significant ($p < 10^{-4}$) showing positive association with the risk of rheumatoid arthritis with odds-ratios of 2.398 (95% CI: 2.187 to 2.629) for the first comparison and 2.760 (95% CI: 2.439 to 3.123) for the second comparison. The Hosmer-*Lemeshow* tests corresponding to the fitted models had *p*-values greater than 0.05, indicating the adequacy of their respective models. The c-statistics for model related to the RA *vs.* CTR comparison was 0.66, and the one for the NARAC-A *vs.* NARAC-C comparison was 0.719. Tables S4 and S5 summarize the results of simple logistic regression analysis based on the overall score variables.

References

1. WTCCC (2007) Genome-wide association study of 14,000 cases of seven common diseases and 3,000 shared controls. Nature 447: 661–678. doi:10.1038/nature05911.

2. Belle G van, Fisher LD, Heagerty PJ, Lumley TS (2004) Biostatistics: A Methodology For the Health Sciences. 2nd ed. Wiley-Interscience. 896 p.

3. Plenge RM, Seielstad M, Padyukov L, Lee AT, Remmers EF, et al. (2007) TRAF1-C5 as a risk locus for rheumatoid arthritis--a genomewide study. N Engl J Med 357: 1199–1209. doi:10.1056/NEJMoa073491.
