# Supplementary material for: A Knowledge-Based Method for Association Studies on Complex Diseases
Source: PLoS One. 2012 Sep 6;7(9):e44162. doi: 10.1371/journal.pone.0044162 (PMC3435396; doi:10.1371/journal.pone.0044162)
Supplement: Results S2 — Description of the results regarding the analysis of Crohn’s disease (CD) dataset. (PDF) [file pone.0044162.s015.pdf]

A Knowledge-based Method for Association Studies on Complex Diseases.

Alireza Nazarian, Heike Sichtig, Alberto Riva

Department of Molecular Genetics and Microbiology & UF Genetics Institute, University of Florida, Gainesville, FL, USA

Supplementary Materials – Results S2

Analysis of the Crohn's Disease (CD) Dataset
Analysis of Test-set and Negative Control Pathways
An initial set of SNPs derived from the 24 pathways under investigation was analyzed by KBAS, using the same strategy used for RA. Subjects in CD group serve as cases and those in 58C and NBS groups consist controls (from [1]). The significance level was set to $6.944x10^{-9}$ according to Bonferroni's correction [2], and the *p*-values resulting from the empirical distribution of the models' test statistic over 100,000 rounds of permutation test was employed as the final criteria to determine the association status of the models. A corrected significance threshold of 0.00104 was applied to interpret the results of permutation test. A model was considered significant if in both CD *vs.* 58C and CD *vs.* NBS comparisons had significant *p*-values, and was considered non-significant if it resulted in non-significant *p*-values in at least one of the two comparisons. In other situations, a model was considered borderline.

Tables S6 and S7 show that the three pathways namely, *B-cell receptor signaling, cytokine-cytokine receptor interaction* and *T-cell receptor signaling* pathways gave rise to multi-SNP models with statistically significant performance in separating the disease group from the two control groups. While they had significant fitness *p*-values comparing CD *vs.* 58C and CD *vs.* NBS ($p < 1.07x10^{-10}$ and $p < 3.22x10^{-10}$ respectively), they were not able to separate the two control groups from each other ($p > 0.0078$). The models related to *B-cell receptor signaling* and *T-cell receptor signaling* pathways also yielded significant *p*-values in permutation tests on both the CD *vs.* 58C and the CD *vs.* NBS comparisons, and are therefore considered strongly associated with Crohn's disease. As the results of permutation test indicates, the model derived from *cytokine-cytokine receptor interaction* pathway was of borderline statistical significance in both CD *vs.* 58C and CD *vs.* NBS comparisons ($p < 0.00121$ and $p < 0.00367$ respectively). This may be suggestive of moderate association between this multi-SNP model and Crohn's disease.

The classifying performance of successful models was also tested by comparing disease group against pooled population of controls (CTR) (see Table S8). Results were interpreted based on the corrected significance thresholds as in the RA *vs.* CTR analysis. In this case, in addition to the three aforementioned ones, six more models proved significant, i.e. those derived from the *antigen processing and presentation,* c*omplement and coagulation cascades, intestinal immune network for IgA production, leukocyte trans-endothelial migration, natural killer cell mediated cytotoxicity and oxidative phosphorylation* pathways. Their fitness *p*-values ranged from $7.40x10^{-9}$ to $1.16x10^{-15}$ and were verified by their respective permutation test *p*-values ranging form less than $8.5x10^{-4}$ to less than

10^{-5}. These pathways may therefore be considered as being associated with Crohn's disease and should be subject to further validation studies in an independent dataset.

Table S9 summarizes the list of SNPs included in the successful models showing association with Crohn's disease, the genes and chromosomes they belong to, their positions on their respective chromosome, and their functional roles. The number of SNPs in these successful models ranged from 4 to 11, which in all cases is less than 1% of the total number of SNPs in the corresponding initial SNP-sets (see Table 1).

SNPs *rs1554286* from *intestinal immune network for IgA production* and *T-cell receptor signaling* pathways and *rs6784820* from *T-cell receptor signaling* pathway are to some extent in LD with *rs3024505* (r^2=0.043 and D'=1) and *rs9858542* (r^2=0.284 and D'=0.965), two previously recognized Crohn's disease-associated SNPs, respectively.

None of the remaining SNPs in these nine models or the genes to which these SNPs belong is among or in linkage disequilibrium with Crohn's disease susceptibility loci detected by genome-wide association studies. Although SNPs *rs2569094* from *antigen processing and presentation*, *rs9724615* from *B-cell receptor signaling*, *rs428060* from c*omplement and coagulation cascades*, *rs2192752* and *rs6876446* from *cytokine-cytokine receptor interaction*, *rs1327473* from *natural killer cell mediated cytotoxicity* and *cytokine-cytokine receptor interaction, rs7555443* and *rs3808917* from *T-cell receptor signaling* and *rs1000984* from *oxidative phosphorylation* pathways are located on chromosome regions less than 1Mbps away from regions which have shown evidence of moderate or strong association with Crohn's disease in previous studies [1,3], there is no evidence of linkage disequilibrium between these nine SNPs and nearby CD-associated SNPs according to HapMap LD data (International HapMap Project: http://hapmap.ncbi.nlm.nih.gov).

As seen in Table S9, the successful models from the c*omplement and coagulation cascades, cytokine-cytokine receptor interaction, intestinal immune network for IgA production, leukocyte trans-endothelial migration, T-cell receptor signaling,* and *oxidative phosphorylation* pathways contained pairs of SNPs on the same chromosome. The large distance between these pairs of SNPs strongly decreases the chance of linkage disequilibrium between them. This is verified by the lack of evidence of linkage disequilibrium between the aforementioned SNP pairs according to HapMap LD data.

Multiple Logistic Regression Analysis

Table S10 summarizes the results obtained from multiple logistic regression analysis conducted for CD *vs.* CTR comparison. The analysis was performed by regressing disease state on the score variables derived from nine successful models demonstrating evidence of association with Crohn's disease (see Tables S8 and S9). The fitted regression model after step-wise model selection procedure contained the score variables from all nine pathways except for those from the *intestinal immune network for IgA production* and *natural killer cell mediated cytotoxicity* pathways. The overall model's *p*-values and the *p*-values of the included covariates were smaller than 10^{-4}. All the included score variables were positively associated with the risk of Crohn's disease and their corresponding odds ratios were in the range of 2.320 to 2.741. The c-statistics for the fitted model was 0.622 and the model's goodness-of-fit was verified by a non-significant Hosmer–Lemeshow test ($p > 0.05$).

Simple Logistic Regression Analysis and Disease Risk-Score Class Diagram

Table S11 summarizes the results obtained from fitting a simple logistic regression model by regressing the overall score variable, computed based on the entire set of 57 SNPs present in the nine CD-associated models (see Tables S8 and S9), on the disease state (CD *vs.* CTR). The *p*-values of the fitted regression model and of the covariates of the overall score variables were statistically significant ($p < 10^{-4}$), and the score variable was in positive association with the risk of disease with odds ratio of 2.506 (95% CI: 2.219 to 2.831). The c-statistics for the fitted model was 0.623 and the Hosmer-*Lemeshow* test was non-significant ($p > 0.05$).

The *Disease risk-Score class* diagram for the CD *vs.* CTR comparison (Figure 3) indicated that the risk of development of Crohn's disease increases with the increase in the score class values. This risk goes up from around 14% for the lowest score class to around 78% for the highest score class.

Comparative Analysis of the Results

Table S12 summarizes the results obtained from the analysis of rheumatoid arthritis and Crohn's disease datasets. While none of the negative control pathways showed association with both rheumatoid arthritis and Crohn's disease, there were multiple immune system related pathways in association with both diseases under investigation. The four models derived from *antigen processing and presentation*, c*omplement and coagulation cascades, intestinal immune network for IgA production,* and *T-cell receptor signaling* pathways were in strong association with rheumatoid arthritis comparing RA *vs.* CTR and NARAC-A *vs.* NARAC-C [4] and with Crohn's disease comparing CD *vs.* CTR. Although models from *leukocyte trans-endothelial migration* and *natural killer cell mediated cytotoxicity* pathways showed strong association with both rheumatoid arthritis and Crohn's disease in the WTCCC dataset, they were in moderate association with RA in the NARAC dataset. Models derived from *B-cell receptor signaling* and *cytokine-cytokine receptor interaction* pathways were in strong association with both diseases only in the WTCCC dataset.

References

1. WTCCC (2007) Genome-wide association study of 14,000 cases of seven common diseases and 3,000 shared controls. Nature 447: 661–678. doi:10.1038/nature05911.

2. Belle G van, Fisher LD, Heagerty PJ, Lumley TS (2004) Biostatistics: A Methodology For the Health Sciences. 2nd ed. Wiley-Interscience. 896 p.

3. Franke A, McGovern DPB, Barrett JC, Wang K, Radford-Smith GL, et al. (2010) Genome-wide meta-analysis increases to 71 the number of confirmed Crohn's disease susceptibility loci. Nat Genet 42: 1118–1125. doi:10.1038/ng.717.

4. Plenge RM, Seielstad M, Padyukov L, Lee AT, Remmers EF, et al. (2007) TRAF1-C5 as a risk locus for rheumatoid arthritis--a genomewide study. N Engl J Med 357: 1199–1209. doi:10.1056/NEJMoa073491.
